# O-GlcNAc transferase in astrocytes modulates depression-related stress susceptibility through glutamatergic synaptic transmission

**DOI:** 10.1172/JCI160016

**Published:** 2023-04-03

**Authors:** Jun Fan, Fang Guo, Ran Mo, Liang-Yu Chen, Jia-Wen Mo, Cheng-Lin Lu, Jing Ren, Qiu-Ling Zhong, Xiao-Jing Kuang, You-Lu Wen, Ting-Ting Gu, Jin-Ming Liu, Shu-Ji Li, Ying-Ying Fang, Cunyou Zhao, Tian-Ming Gao, Xiong Cao

**Affiliations:** 1 Key Laboratory of Mental Health of the Ministry of Education, Guangdong–Hong Kong–Macao Greater Bay Area Center for Brain Science and Brain-Inspired Intelligence, Guangdong Province Key Laboratory of Psychiatric Disorders, Department of Neurobiology, School of Basic Medical Sciences, Southern Medical University, Guangzhou, Guangdong, China.; 2 Department of Anesthesia, Guangzhou Women and Children’s Medical Center, Guangzhou Medical University, Guangdong Provincial Clinical Research Center for Child Health, Guangzhou, Guangdong, China.; 3 Department of Psychology, School of Public Health, Southern Medical University, Guangzhou, Guangdong, China.; 4 Department of Psychology and Behavior, Guangdong 999 Brain Hospital, Institute for Brain Research and Rehabilitation, South China Normal University, Guangzhou, Guangdong, China.; 5 Department of Medical Genetics, School of Basic Medical Sciences, and Guangdong Technology and Engineering Research Center for Molecular Diagnostics of Human Genetic Diseases,; 6 Department of Oncology, Nanfang Hospital, and; 7 Microbiome Medicine Center, Department of Laboratory Medicine, Zhujiang Hospital Southern Medical University, Guangzhou, Guangdong, China.

**Keywords:** Neuroscience, Behavior, Depression

## Abstract

Major depressive disorder is a common and devastating psychiatric disease, and the prevalence and burden are substantially increasing worldwide. Multiple studies of depression patients have implicated glucose metabolic dysfunction in the pathophysiology of depression. However, the molecular mechanisms by which glucose and related metabolic pathways modulate depressive-like behaviors are largely uncharacterized. Uridine diphosphate *N*-acetylglucosamine (UDP-GlcNAc) is a glucose metabolite with pivotal functions as a donor molecule for O-GlcNAcylation. O-GlcNAc transferase (OGT), a key enzyme in protein O-GlcNAcylation, catalyzes protein posttranslational modification by O-GlcNAc and acts as a stress sensor. Here, we show that *Ogt* mRNA was increased in depression patients and that astroglial OGT expression was specifically upregulated in the medial prefrontal cortex (mPFC) of susceptible mice after chronic social-defeat stress. The selective deletion of astrocytic OGT resulted in antidepressant-like effects, and moreover, astrocytic OGT in the mPFC bidirectionally regulated vulnerability to social stress. Furthermore, OGT modulated glutamatergic synaptic transmission through O-GlcNAcylation of glutamate transporter-1 (GLT-1) in astrocytes. OGT astrocyte–specific knockout preserved the neuronal morphology atrophy and Ca^2+^ activity deficits caused by chronic stress and resulted in antidepressant effects. Our study reveals that astrocytic OGT in the mPFC regulates depressive-like behaviors through the O-GlcNAcylation of GLT-1 and could be a potential target for antidepressants.

## Graphical abstract

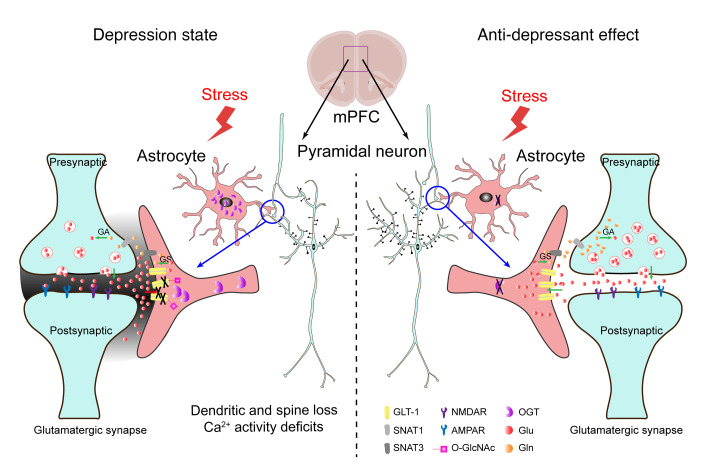

## Introduction

The lifetime prevalence (~17%), disability (as a leading cause), and socioeconomic burden associated with major depressive disorder (MDD) render it one of the most common and devastating psychiatric illnesses worldwide (1). Moreover, the prevalence and burden of MDD exhibited a substantial increase during the COVID-19 pandemic (2). In addition to evidence from patients with depression, especially medication-resistant depression, PET scanning was used to reveal pronounced glucose hypometabolism in the medial prefrontal cortex (mPFC) (3). In addition, antidepressant treatment has been associated with significant increases in metabolic activity in the mPFC (3–5). However, how glucose-related metabolic pathways are involved in the pathogenesis of depression remains largely unexplored.

Uridine diphosphate–*N*-acetylglucosamine (UDP-GlcNAc) is a glucose metabolite in the hexosamine biosynthetic pathway (HBP) with pivotal functions as a donor molecule for transferring GlcNAc to proteins (O-GlcNAcylation) (6). O-GlcNAcylation is a dynamic posttranslational modification (PTM) that fine-tunes multiple processes governing fundamental cellular processes, such as signal transduction, transcription, translation, and proteasomal degradation (6–8). O-GlcNAc transferase (OGT), a key enzyme in protein O-GlcNAcylation, catalyzes the covalent attachment of single O-GlyNAc to the serine or threonine residues of proteins (7). OGT in the placenta plays an important role in depressive and anxiety-like behaviors and memory impairment induced by prenatal stress and exhibits sex differences in offspring (9–11). Although OGT is ubiquitously expressed, cytosolic OGT activity in the brain is 10 times higher than that in muscle, adipose tissue, the heart, and the liver (6). In the brain, OGT has been shown to be involved in neuronal development, adult neurogenesis, neuron survival, and feeding behaviors (12–16). Stress is an important precursor for depression (17, 18), and OGT acts as a nutrient and stress sensor (6, 19–22). However, the central role of OGT in stress-related emotions and mood disorders is almost completely unknown.

In the mPFC, neurons communicate with each other and serve as a central hub in the emotional circuits of the brain by synapses, a tripartite structure consisting of the axon terminal of one neuron, the postsynaptic membrane of another neuron, and the surrounding glial cell processes (23). Astrocytes, the majority of glial cells, ensheath most excitatory synapses, regulate neuronal transmission, and participate in the regulation of the stress response (24). Studies in both depressed humans and animals have demonstrated an obvious reduction in the density of glial cells in the mPFC (24–26). Dysfunction of astrocytes contributes to depressive-like behaviors (25, 27–29). Astrocytes are perfectly positioned to balance glucose metabolism in the brain because their end-feet have rosette-like structures lying on the blood vessel wall, which allows the direct exchange of metabolites between capillaries and nerve terminals (30). Astrocytes are recognized as active players in brain energy delivery, production, utilization, and storage and provide substrates for many biological processes (31, 32). Astrocyte-derived ATP and lactate, the major products of glucose metabolism, contribute to the modulation of depressive-like behaviors (27, 33, 34). However, knowledge regarding astrocyte-related OGT/O-GlcNAcylation in MDD is limited.

Here, we show that a metabolic and stress sensor, OGT, was enriched in astrocytes and neurons. OGT in astrocytes was upregulated in a depression model and was sufficient to bidirectionally regulate susceptibility to stress in the mPFC, but not in neurons. Astrocytic OGT in the mPFC modulated glutamatergic transmission through the O-GlcNAcylation of glutamate transporter-1 (GLT-1). Importantly, this action preserved neuronal morphology and calcium activity under chronic stress. These results suggest, for what we believe is the first time, that astrocytic OGT can protect mPFC pyramidal neurons from social stress by regulating the O-GlcNAcylation of GLT-1 and indicate the essential role of OGT in stress-induced depression.

## Results

### Astrocytic OGT is upregulated in the mPFC after CSDS.

**Figure 1:**
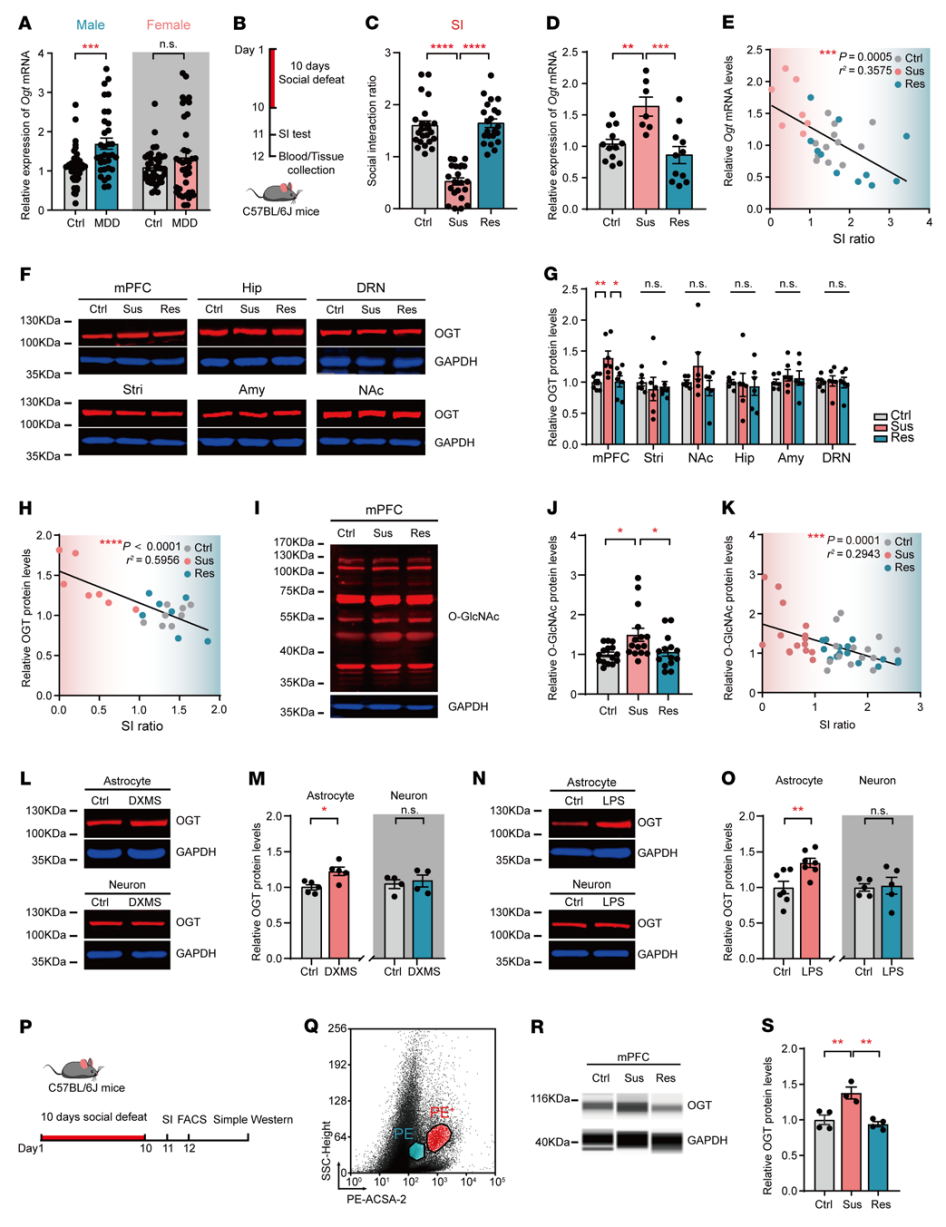
Astrocytic OGT is increased in the mPFC after CSDS. (**A**) *Ogt* mRNA levels in peripheral blood from MDD patients and healthy controls by sex. MDD patients (33 males and 37 females); healthy control subjects (38 males and 39 females). (**B**) Paradigms of 10 days of CSDS and SI tests. (**C**) C57BL/6J mice were divided into the Sus and Res subgroups according to the SI ratio after CSDS. *n* = 23 (control [Ctrl]); *n* = 22 (Sus); *n* = 22 (Res). (**D**) *Ogt* mRNA expression in blood from C57BL/6J mice after CSDS. (**E**) Relationship between *Ogt* levels and SI ratio, *P* = 0.0005. *n* = 12 (control); *n* = 7 (Sus); *n* = 11 (Res). (**F** and **G**) OGT protein levels in mood-related brain regions. *n* = 8 (Ctrl); *n* = 7 (Sus); *n* = 7 (Res) mice in the mPFC; *n* = 6 mice in the Stri, NAc, Hip, Amy, and DRN. (**H**) Relationship between OGT protein levels in the mPFC and SI, *P* < 0.0001. *n* = 8 (Ctrl); *n* = 7 (Sus); *n* = 7 (Res). (**I** and **J**) Protein O-GlcNAc levels in the mPFC after CSDS. *n* = 15 (Ctrl); *n* = 15 (Sus); *n* = 14 (Res) mice. (**K**) Pearson’s correlation analysis between total O-GlcNAc expression and SI, *P* = 0.0001. *n* = 15 (Ctrl); *n* = 15 (Sus); *n* = 14 (Res). (**L** and **M**) OGT protein expression in primary cultured astrocytes and neurons treated with DXMS. *n* = 5 in astrocytes; *n* = 4 in neurons. (**N** and **O**) OGT protein expression in primary cultured astrocytes and neurons treated with LPS. *n* = 7 in astrocytes; *n* = 5 in neurons. **(P** and **Q**) Analysis of astrocytes sorted by FACS in the mPFC following CSDS. (**R** and **S**) Astrocytic OGT expression in the mPFC after CSDS. *n* = 4 (Ctrl); *n* = 3 (Sus); *n* = 4 (Res) trials involving 6–8 mice each. Data are represented as mean ± SEM. Two-sided unpaired *t* test (**M** and **O**) or Mann-Whitney *U* test (**A**) for 2 groups; 1-way ANOVA followed by Bonferroni’s test for multiple comparisons (**C**, **D**, **G**, **J**, and **S**). *P < 0.05; **P < 0.01; ***P < 0.001; ****P < 0.0001. See Supplemental Data Set 1 for statistical details.

To explore whether O-GlcNAcylation is involved in depression, we examined the *Ogt* mRNA levels in peripheral blood from MDD patients (33 males and 38 females) and healthy control subjects (38 males and 39 females) by real-time quantitative PCR (qPCR). The demographic and clinical characteristics of the subjects are listed in Supplemental Tables 1–3 (supplemental material available one with this article; https://doi.org/10.1172/JCI160016DS1). The data analysis showed that *Ogt* mRNA was significantly increased in blood from male MDD patients compared with that in age-matched normal control subjects, while no significant difference was observed in female MDD patients (Figure 1A). Furthermore, we reanalyzed data sets published in the NCBI’s Gene Expression Omnibus database involving RNA-Seq (GEO GSE80655) (35) and microarray (GEO GSE54570) (36) of prefrontal cortex tissue from MDD patients and controls, and the results also showed that *Ogt* was increased in dorsolateral PFC (DLPFC) (Brodmann area 9 [BA9]) of MDD patients compared with control subjects (Supplemental Figure 1, A–D). Then, we used the chronic social-defeat stress (CSDS) model, which mimics several psychopathological dimensions of depression (37) (Figure 1B and Supplemental Figure 2A), and the adult C57BL/6J mice subjected to CSDS were separated into susceptible (Sus) and resilient (Res) subpopulations by the social interaction (SI) ratio (38) (Figure 1, B and C). Peripheral blood was collected, and *Ogt* mRNA expression in each group was measured by qPCR; the levels of *Ogt* mRNA in the Sus group after the CSDS paradigm were significantly increased compared with those in the control or Res groups (Figure 1D). Additionally, the levels of *Ogt* mRNA in blood were negatively correlated with the SI ratio (Pearson’s correlation coefficient, *r^2^* = 0.3575; *P* = 0.0005) (Figure 1E). Tissues from several mood-related brain regions, including the mPFC, nucleus accumbens (NAc), amygdala (Amy), striatum (Stri), hippocampus (Hip), and dorsal raphe nucleus (DRN), were collected after CSDS. Western blot analysis showed that OGT was significantly upregulated in the mPFC in the Sus group after CSDS compared with that in the control and Res groups (Figure 1, F and G). Meanwhile, the OGT protein levels in the mPFC and SI ratio were negatively correlated (Pearson’s correlation coefficient, *r^2^* = 0.5956; *P* < 0.0001) (Figure 1H). However, no obvious change was observed in the NAc, Amy, Stri, Hip, or DRN (Figure 1, F and G). Furthermore, the increased expression levels of total O-GlcNAcylation in the mPFC were confirmed in the Sus mice (Figure 1, I and J). As shown in Figure 1K, a negative correlation was observed between the O-GlcNAcylation protein levels and the SI ratio (Pearson’s correlation coefficient, *r^2^* = 0.2943; *P =* 0.0001) (Figure 1K). However, after 3 days of subthreshold social-defeat stress (SSDS), the protein levels of OGT and O-GlcNAc were not altered in the mPFC (Supplemental Figure 2, B–D).

OGT is ubiquitously expressed in the adult brain, including in 2 major cell types, neurons and astrocytes (39) (Supplemental Figure 2, E–I). To determine whether neurons or astrocytes contribute to the increased OGT levels, primary cultured astrocytes and neurons were treated with dexamethasone (DXMS) (1 μM) or lipopolysaccharide (LPS) (1 μg ml^–1^) to mimic the stress condition in vitro. Western blot analysis showed that OGT was significantly increased in the cultured astrocytes, but not neurons, after the DXMS or LPS treatment (Figure 1, L–O). To confirm the change in the astrocytic OGT levels in vivo, astrocytes in the mPFC were collected by FACS after CSDS (Figure 1, P and Q, and Supplemental Figure 2, J and K). Simple Western blot analysis also showed that the astrocytic OGT levels were significantly increased in the mPFC in the Sus mice compared with those in the control and Res mice (Figure 1, R and S). Taken together, these results suggest that astrocytic OGT may be involved in depressive-like behaviors.

### Selective deletion of OGT in astrocytes results in antidepressant-like effects.

**Figure 2:**
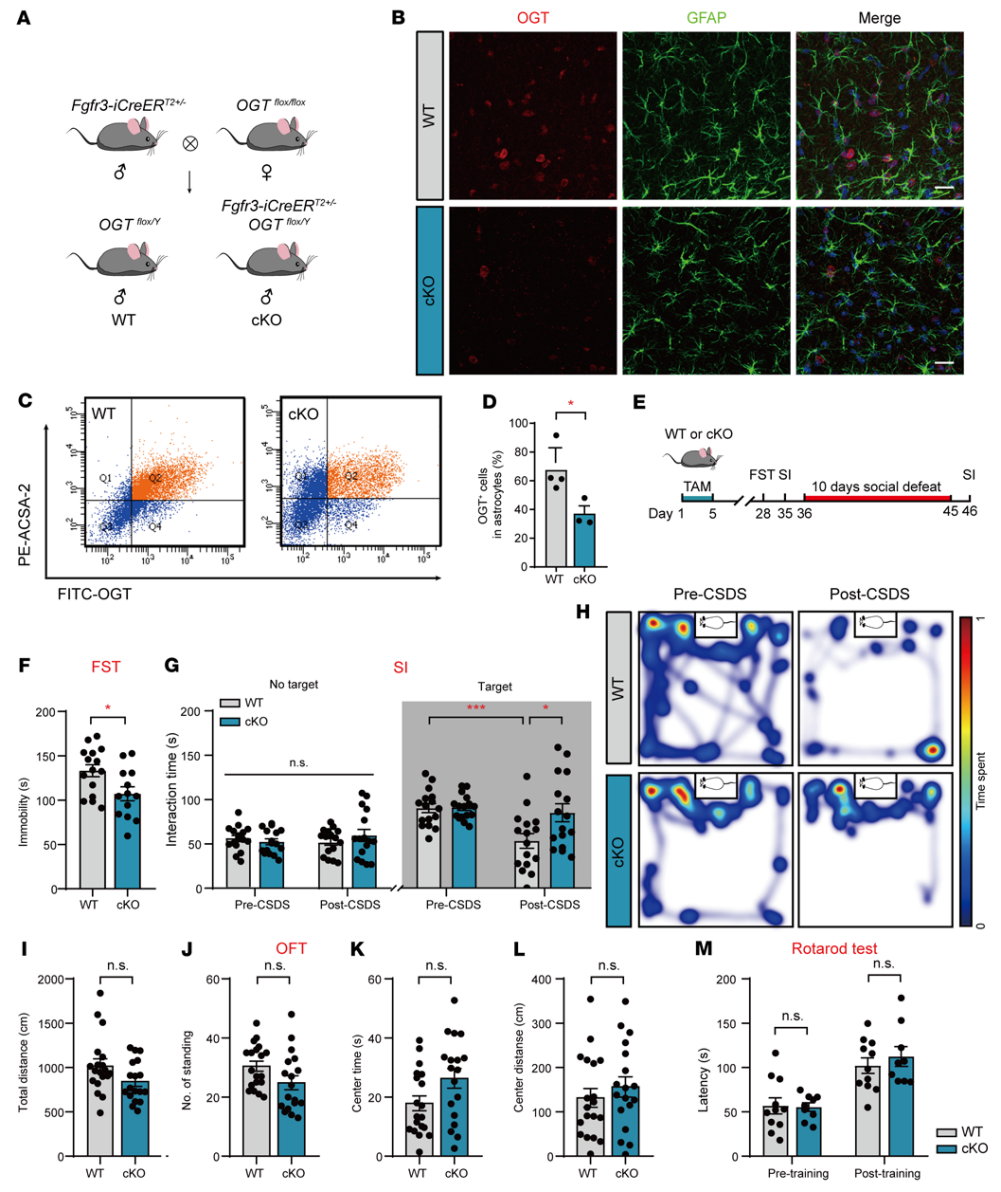
Deletion of astrocytic OGT results in antidepressant-like effects. (**A**) Generation of OGT-cKO mice by crossing *OGT^fl/fl^* lines with *Fgfr3-iCreER^T2^* lines. (**B**) Double-immunofluorescence staining of OGT (red) with GFAP (green) in cKO and WT mice. Scale bars: 20 μm. (**C** and **D**) FACS images and quantification of OGT-positive astrocytes from cKO and WT mice. *n* = 4 (WT); *n* = 3 (cKO) mice. (**E**) Paradigms of behavioral analysis of cKO mice and littermate controls using the FST and CSDS. (**F**) Immobility time of cKO and WT mice in the FST. *n* = 15 (WT); *n* = 13 (cKO) mice. (**G** and **H**) SI time in the absence or presence of a social target (**G**). *n* = 16 (WT); *n* = 16 (cKO) mice. Representative heatmaps (**H**) of WT (top) and cKO (bottom) mice in the presence of social targets before CSDS (left) and after CSDS (right). (**I**–**L**) OFT of *Fgfr3-iCreER^T2^; OGT^fl/Y^* (cKO) and WT mice. *n* = 19 (WT); *n* = 18 (cKO). (**M**) Rotarod test of cKO and WT mice. *n* = 15 (WT); *n* = 13 (cKO) mice. Data are represented as mean ± SEM. Two-sided unpaired *t* test (**D**, **F**, and **I**–**L**); 2-way ANOVA with Bonferroni’s multiple-comparisons test (**G** and **M**). *P < 0.05; ***P < 0.001. See Supplemental Data Set 1 for statistical details.

The *Ogt* gene localizes at *Xq13*, and global OGT deletion results in embryonic lethality (40). To investigate potential astrocyte- or neuron-specific effects associated with depressive-related phenotypes derived from *Ogt* gene deletion, we generated astrocyte-specific (Figure 2A) (41) and neuron-specific (Supplemental Figure 5A) conditional *Ogt* deletion mouse lines by crossing the floxed OGT allele with *Fgfr3-iCreER^T2^* or *CaMKIIα-creER^T2^* transgenic lines. Consistent with a previous report (15), the OGT neuron-specific conditional knockout (cKO) mice showed an increased growth rate and body size after tamoxifen (TAM) injection to induce the expression of Cre recombinase (Supplemental Figure 5, B–E). The astrocyte-specific cKO animals exhibited normal growth rates and body sizes (Supplemental Figure 3, A–D). Additionally, the deletion of OGT in astrocytes did not lead to gross anatomical changes in the brain or the densities of astrocytes and neurons (Supplemental Figure 3, E–J) (41). To confirm an astrocyte-specific OGT reduction in the *Fgfr3-iCreER^T2^; OGT^fl/Y^* mice, we employed a specific OGT antibody and costained with glial fibrillary acidic protein (GFAP), a marker of astrocytes. Immunofluorescence revealed that the GFAP-labeled astrocytes featured attenuated OGT staining (Figure 2B). Then, FACS was performed in astrocyte cKO animals, and the number of OGT-positive astrocytes was reduced by approximately 50% in the cKO mice compared with that in the WT littermate mice (Figure 2, C and D).

Next, we investigated the behavioral performances of the OGT-cKO mice and littermate controls (Figure 2E). In the forced swimming test (FST), the astrocyte-specific cKO mice showed a significant decrease in immobility duration (Figure 2F). In the CSDS experiments, the astrocyte-specific cKO and WT mice showed interaction times indistinguishable from those of a CD1 aggressor mouse before CSDS. After 10 days of CSDS, the WT mice presented depressive-like behaviors, as indicated by a significantly reduced interaction time with aggressors, whereas the development of social avoidance was inhibited in the astrocyte-specific cKO mice (Figure 2, G and H).

The astrocyte-specific cKO mice displayed slightly reduced general locomotion in the open-field test (OFT), but these reductions were not significant (Figure 2, I–L); moreover, no differences were observed in motor coordination in the rotarod test (Figure 2M). In the light-dark (LD) box test, the astrocyte-specific cKO mice showed significantly decreased dark entries without influencing the dark duration and entries (Supplemental Figure 4, A–D), and no behavioral differences were observed in the elevated plus maze (EPM) (Supplemental Figure 4E), novelty suppressed feeding (NSF) test (Supplemental Figure 4, F and G), or central time in the OFT (Figure 2K). The astrocyte-specific cKO mice did not exhibit obvious differences in the T maze (Supplemental Figure 4H) or SI tests (Supplemental Figure 4I), indicating that the astroglial deletion of OGT had no effects on forced locomotor activity, anxiety, learning, memory, or social novelty. However, the neuron-specific OGT-cKO mice did not exhibit a depressive-like phenotype in the FST (Supplemental Figure 5N), and there was a slight decrease in dark entries in the LD test and center distance in the OFT (Supplemental Figure 5, F–N). Together, these data indicate that the selective knockout of OGT in adult astrocytes produced antidepressant-like effects.

### Astrocytic OGT in the mPFC bidirectionally regulates susceptibility to social stress.

**Figure 3:**
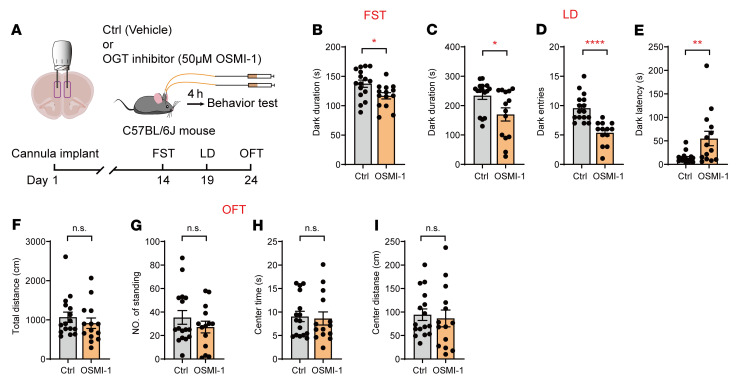
Inhibition of OGT in the mPFC produces antidepressant effects. (**A**) Schematic of bilateral cannula implantation and behavioral tests. C57BL/6J mice were infused with OSMI-1 (OGT inhibitor, 50 μM) or vehicle 4 hours before the behavioral test. (**B**) FST of C57BL/6J mice infused with OSMI-1 or vehicle. *n* = 16 (Ctrl); *n* = 14 (OSMI-1) mice. (**C**–**E**) LD test of C57BL/6J mice infused with OSMI-1 or vehicle. (**F**–**I**) OFT of C57BL/6J mice infused with OSMI-1 or vehicle. *n* = 16 (Ctrl); *n* = 14 (OSMI-1) mice. Data are represented as mean ± SEM. Two-sided unpaired *t* test (**B**–**D** and **F**–**I**); Mann-Whitney *U* test (**E**) for 2-group comparisons. *P < 0.05; **P < 0.01; ****P < 0.0001. See Supplemental Data Set 1 for statistical details.

Our previous data suggested that astrocytic dysfunction is involved in depressive-like behaviors and that OGT levels are selectively increased in mPFC astrocytes in Sus mice. To test the brain region–specific effect of OGT on behaviors, we first employed cannula infusion of an OGT inhibitor into the mPFC. Therefore, a bilateral cannula was implanted above the mPFC core of adult C57BL/6J mice, and behavioral tests were conducted after recovery (Figure 3A). After the infusion of OSMI-1 (50 μM) (42, 43), an OGT inhibitor, mice exhibited a significantly decreased immobility time compared with those in the control group in the FST (Figure 3B). Moreover, the inhibition of OGT in the mPFC was sufficient to induce reduced duration and entries in the dark box and increased dark latency in the LD test (Figure 3, C–E) without affecting locomotor activity in the OFT (Figure 3, F–I).

**Figure 4:**
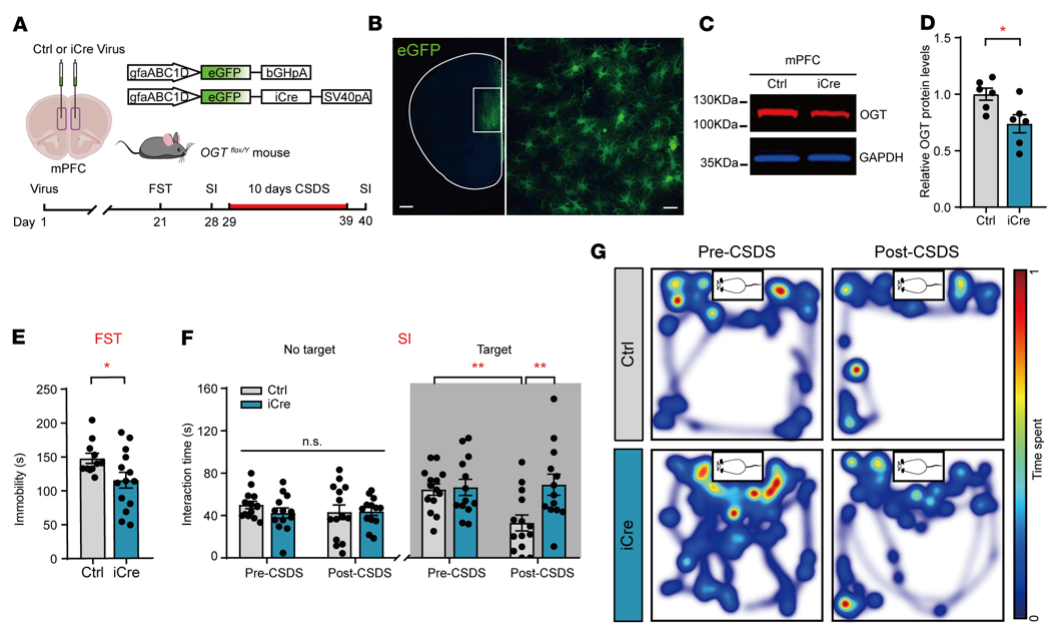
Astrocyte-specific deletion of OGT in the mPFC results in antidepressant effects. (**A**) Schematic of the AAV vectors engineered to specifically knock down astrocytic OGT in the mPFC and behavioral test paradigms. (**B**) Representative images of injection sites in the mPFC of *OGT^fl/Y^* mice. Scale bars: 500 μm (right, magnified view of the left image): 20 μm (left). (**C** and **D**) Western blot images (**C**) and quantification (**D**) of OGT in the mPFC of *OGT^fl/Y^* mice. *n* = 6 (Ctrl); *n* = 6 (iCre) mice. (**E**) Immobility time of mice with selective OGT knockdown in the FST. *n* = 11 (Ctrl); *n* = 14 (iCre) mice. (**F**) SI time in the absence or presence of social targets before and after CSDS. *n* = 14 (Ctrl); *n* = 13 (iCre) mice. (**G**) Representative heatmaps of selective OGT-knockdown mice in the presence of social targets before CSDS (left) and after CSDS (right). Data are represented as mean ± SEM. Two-sided unpaired *t* test (**D** and **E**); 2-way ANOVA with Bonferroni’s multiple-comparisons test (**F**). *P < 0.05; **P < 0.01. See Supplemental Data Set 1 for statistical details.

To determine whether OGT in mPFC astrocytes is required for the antidepressant-like effects, we specifically knocked out OGT in the mPFC of adult *OGT^fl/Y^* mice using bilateral injection of adeno-associated virus serotype 2/9 (AAV2/9), which preferentially targets astrocytes along with the human GFAP (gfaABC1D) promoter (28), to deliver Cre recombinase (AAV-gfaABC1D-GFP-iCre) or EGFP alone (AAV-gfaABC1D-EGFP) as a control (Figure 4A). The AAV2/9-mediated viral transfection led to a reduced expression of OGT in astrocytes in the mPFC (Figure 4, B–D). The AAV-gfaABC1D-iCre–infected mice also exhibited antidepressant-like phenotypes, a decreased immobility time in the FST (Figure 4E), and increased interaction times with CD1 after 10 days of CSDS, while the control mice developed social avoidance (Figure 4, F and G). The viral infection did not affect performance in the OFT, LD test, or EPM (Supplemental Figure 6, A–H). To determine whether some other nonspecific mechanism might be involved, adult *OGT^fl/Y^* mice were bilaterally injected with gABC1D-iCre or control virus in the ventral Stri (NAc) (Supplemental Figure 7, A and B), which was also involved in the regulation of depressive-like behaviors (44). Western blot analysis confirmed the decrease in OGT protein levels (Supplemental Figure 7, C and D). In the behavioral tests, no change was observed in the OFT, FST, LD, or SI (Supplemental Figure 7, E–M). These results suggest that a reduced astrocytic OGT level in the mPFC was sufficient to produce antidepressant-like effects.

**Figure 5:**
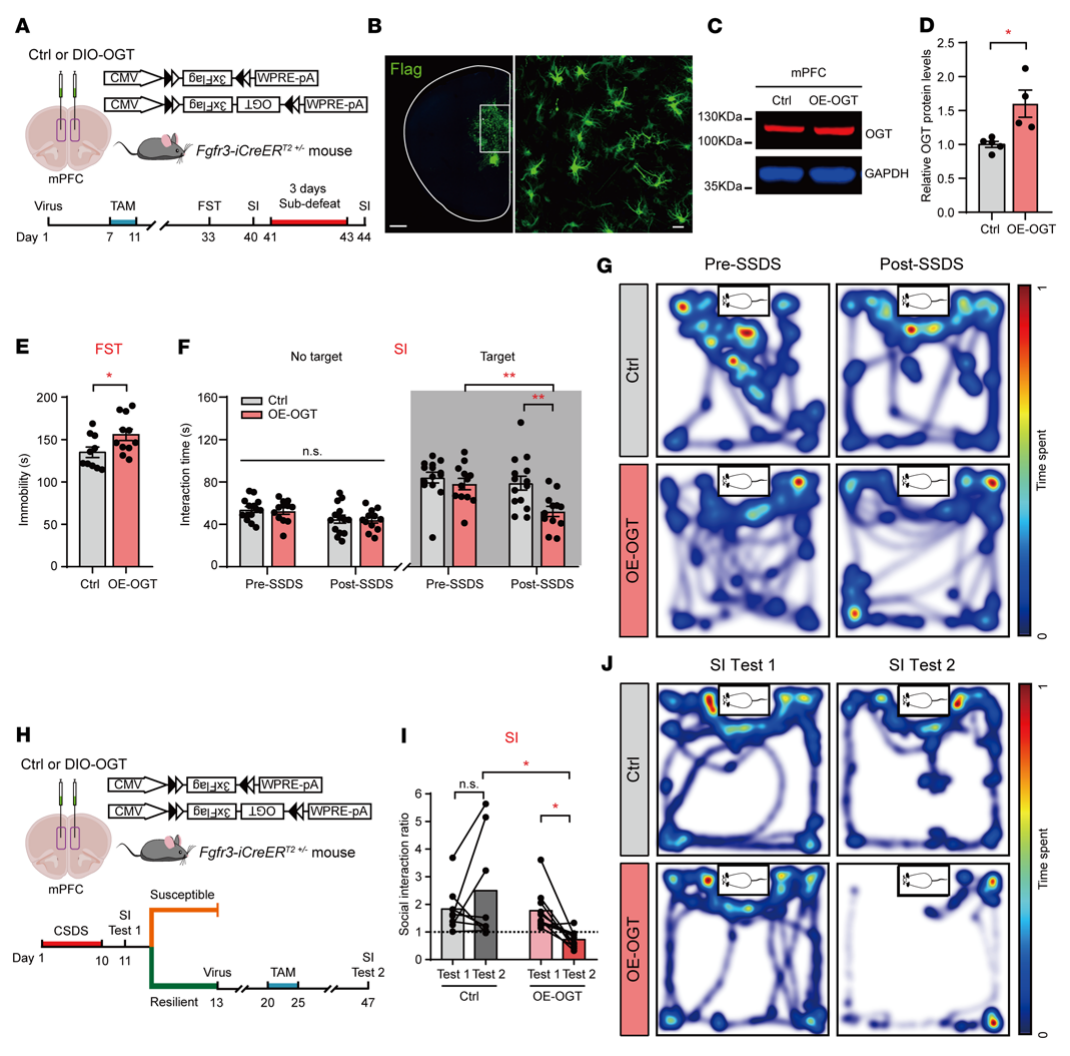
OE of astrocytic OGT in the mPFC increases stress susceptibility. (**A**) Schematic of the AAV vectors engineered to specifically OE astrocytic OGT and behavioral test paradigms. (**B**) Representative images of the injection sites in the mPFC of *Fgfr3-iCreER^T2^* mice. Scale bars: 500 μm (right, magnified view of the left image); 20 μm (left). (**C** and **D**) Western blot images (**C**) and quantification (**D**) of OGT in the mPFC of *Fgfr3-iCreER^T2^* mice. *n* = 5 (Ctrl); *n* = 4 (OE) mice. (**E**) Immobility time of mice with selective OGT OE in the FST. *n* = 10 (Ctrl); *n* = 11 (OE) mice. (**F**) SI time in the absence or presence of social targets before and after CSDS. *n* = 14 (Ctrl); *n* = 12 (OE) mice. (**G**) Representative heatmaps of selective OGT-OE mice in the presence of social targets before CSDS (left) and after CSDS (right). (**H**) Schematic of the AAV vectors engineered to specifically OE astrocytic OGT in the mPFC of Res mice following 10 days of CSDS and behavioral test paradigms. (**I**) SI ratio in the SI test before (SI test 1) and after virus expression (SI test 2). *n* = 8 (Ctrl); *n* = 10 (iCre) mice. (**J**) Representative heatmaps of OE astrocytic OGT in the mPFC of Res mice following CSDS SI test 1 (left) and SI test 2 (right). Data are represented as mean ± SEM. Two-sided unpaired *t* test (**E**) or Mann-Whitney *U* test (**D**) for 2-group comparisons; 2-way ANOVA with Bonferroni’s multiple-comparisons test (**F** and **I**). *P < 0.05; **P < 0.01. See Supplemental Data Set 1 for the statistical details.

To assess the consequences of OGT overexpression (OE) in mPFC astrocytes, we infused AAV-DIO-OGT-3×Flag (DIO-OGT) or AAV-DIO-3×Flag (DIO-Ctrl) virus into the mPFC of *Fgfr3-iCreER^T2^* mice (Figure 5A). Confocal images showed that the viruses were expressed in the astrocytes in the mPFC (Figure 5B). Western blot analysis confirmed the increased OGT levels in the mPFC (Figure 5, C and D). In the behavioral tests, *Fgfr3-iCreER^T2^* mice with DIO-OGT infection in the mPFC presented significantly increased immobility time in the FST (Figure 5E). To assess stress susceptibility, we adopted a 3-day SSDS model (45). At baseline, there was no difference between the AAV-DIO-OGT–infected and control mice. Notably, following 3 days of SSDS, the mice with AAV-DIO-OGT infection spent less time in the interaction zone than the control mice (Figure 5, F and G). No behavioral changes were observed in the OFT, LD test, or EPM (Supplemental Figure 6, I–P). These data suggest that astrocytic OGT upregulation in the mPFC increases stress susceptibility.

So that we could better understand the regulation of astrocytic OGT in the mPFC, 8-week-old *Fgfr-iCreERT^+/–^* male mice were subjected to 10 days of CSDS, and all Res mice were selected after the SI test (Figure 5H). The Res mice were randomly divided into the control and OE-OGT groups by bilateral injection of DIO-Ctrl or DIO-OGT virus in the mPFC (Figure 5H). TAM was used to induce the expression of Cre recombinase 1 week after the virus injection (Figure 5H). Confocal images and Western blot analysis confirmed the increased levels of OGT in the astrocytes in the mPFC (Supplemental Figure 8, A–C). In the SI test, the Res mice injected with control virus still exhibited greater than 1 in the SI ratio and remained resilient to social stress (Figure 5, I and J). However, the OE of astrocytic OGT in the mPFC was sufficient to render the Res mice susceptible to stress, as indicated by the significantly reduced SI ratio with aggressors (Figure 5, I and J). Taken together, these results suggest that OGT in mPFC astrocytes bidirectionally regulates susceptibility to social stress.

### Identification and enrichment of O-GlcNAcylation proteins in the mPFC.

**Figure 6:**
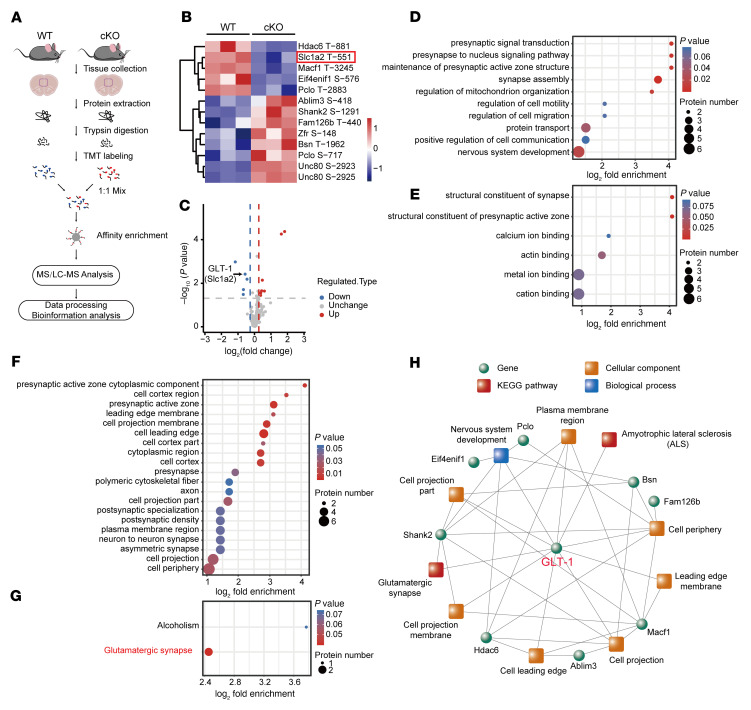
Identification and enrichment of O-GlcNAcylation proteins in the mPFC. (**A**) Schematic of the proteomics analysis of O-GlcNAcylation in the mPFC. (**B** and **C**) Heatmap (**B**) and volcano plots (**C**) of differentially modified O-GlcNAcylation protein sites. LC, liquid chromatography. (**D**–**F**) GO enrichment of differentially modified proteins, including the biological process (**D**), molecular function (**E**), and cellular compartment (**F**) categories. Two-tailed Fisher’s exact test and corrected *P* < 0.05 were considered significant. (**G**) KEGG enrichment of differentially modified proteins. Two-tailed Fisher’s exact test and corrected *P* < 0.05 were considered significant. (**H**) Coexpression network of differentially modified O-GlcNAcylation proteins and GO and KEGG pathways.

To investigate the underlying mechanism by which astrocytic OGT regulates depressive-like behavior, mPFC tissues were collected from astrocyte-specific cKO and WT mice for proteomics analysis of O-GlcNAcylation (Figure 6A). In total, 38,992 spectra were examined, and 322 O-GlcNAc modification sites from 190 proteins were identified (Supplemental Figure 9A). Most identified proteins had 1 to 2 O-GlcNAcylation sites (Supplemental Figure 9, A and B). In total, 211 of these O-GlcNAcylation sites were quantified in both groups (Supplemental Figure 9A). Thirteen O-GlcNAcylation sites from 12 different proteins were differentially expressed (Figure 6, B and C). Gene Ontology (GO) and Kyoto Encyclopedia of Genes and Genomes (KEGG) pathways were analyzed according to these differentially expressed O-GlcNAcylation sites. Three different categories, including biological process, cellular compartment, and molecular function, were enriched. In the biological process category, synaptic-related processes were enriched, including presynaptic signal transduction, the presynapse-to-nucleus signaling pathway, and synapse assembly (Figure 6D). The structural constituents of synapses and structural constituents of the presynaptic active zone were enriched in the cellular compartment category (Figure 6E). In the molecular function category, synaptic functions, such as presynapse, presynaptic active zones, and postsynaptic specialization, were also enriched (Figure 6F). In GO enrichment analysis, data displayed a strong relation to synaptic functions. KEGG enrichment analysis revealed that glutamatergic synapses were significantly enriched (Figure 6G). Glutamate is the most abundant excitatory neurotransmitter in the brain. Slc1a2 (also known as GLT-1 and EAAT2), a glutamate transporter mostly expressed on astrocytes (46), can clear glutamate at excitatory synapses and maintain the extracellular glutamate concentrations at low values by degrading glutamate into glutamine to prevent glutamate excitotoxicity due to the lack of extracellular enzymes in neurons (47). The O-GlcNAcylation proteomics analysis showed that the O-GlcNAcylation of GLT-1 was significantly decreased in the mPFC of the cKO mice compared with that in the WT controls (Figure 6, B, C, and H). These data suggest that astrocytic OGT may be involved in glutamatergic synapses via the O-GlcNAcylation of GLT-1.

### OGT regulates glutamate signaling in the mPFC through GLT-1 O-GlcNAcylation.

**Figure 7:**
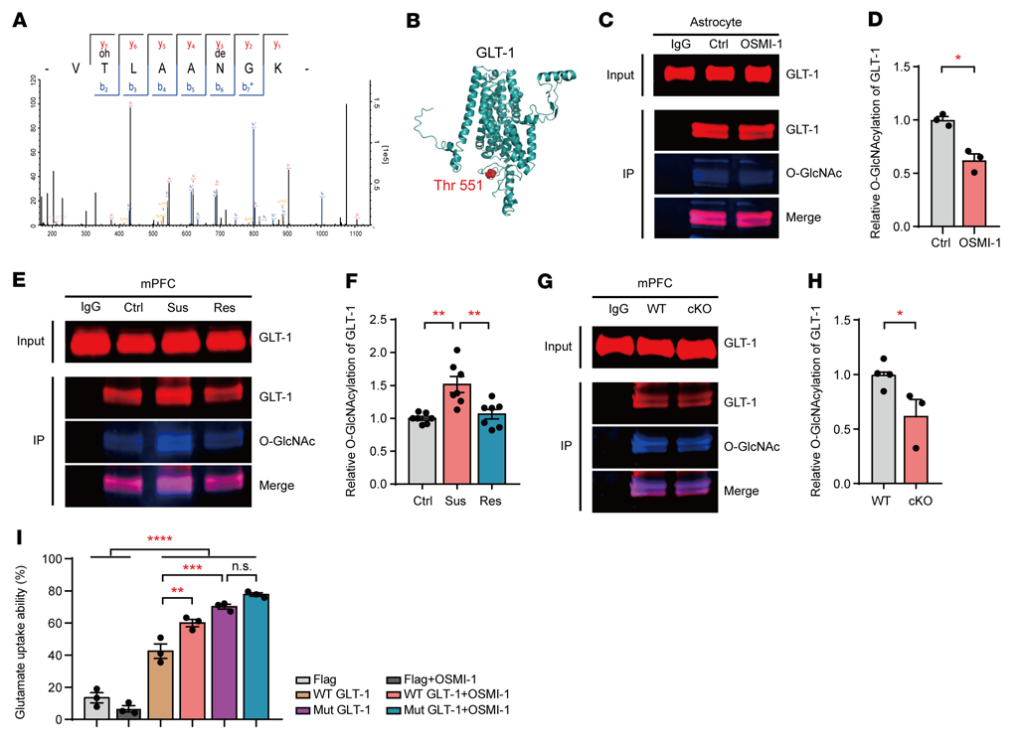
OGT in astrocytes modulates glutamate uptake ability through the O-GlcNAcylation of GLT-1. (**A**) Spectrum of GLT-1 showing that Thr-551 was modified by O-GlcNAcylation. (**B**) Structure of GLT-1 was predicted by RaptorX-Contact and visualized using PyMOL, version 1.3. Thr-551 is labeled with a red sphere. (**C** and **D**) Immunoprecipitation of GLT-1 from cultured astrocytes to analyze the consequences of OSMI-1 (OGT inhibitor, 50 μM) on the O-GlcNAcylation of GLT-1. Precipitation with rabbit IgG was used as a negative control. Input lanes were loaded with 5% of the tissue lysates used for IP. *n* = 3 (Ctrl); *n* = 3 (OSMI-1). (**E** and **F**) GLT-1 was immunoprecipitated from the mPFC of control, Sus, and Res mice after CSDS, and the O-GlcNAcylation of GLT-1 was quantified. *n* = 7 (Ctrl); *n* = 7 (Sus); *n* = 7 (Res). (**G** and **H**) O-GlcNAcylation of GLT-1 was quantified in the mPFC of OGT-cKO and WT mice. *n* = 4 (WT); *n* = 3 (cKO). (**I**) Glutamate-uptake ability in medium of H293T cells transfected with mutant GLT-1 and control plasmids treated with or without the OGT inhibitor OSMI-1. *n* = 3 per group. Data are represented as mean ± SEM. Two-sided unpaired *t* test (**D** and **H**); 1-way ANOVA with Bonferroni’s multiple-comparisons test (**F** and **I**). *P < 0.05; **P < 0.01; ***P < 0.001; ****P < 0.0001. See Supplemental Data Set 1 for statistical details.

To investigate the role of OGT in the O-GlcNAcylation of GLT-1, we first conducted a mass spectrometry (MS) analysis. The MS/MS spectra of GLT-1 showed that Thr-551 was modified by O-GlcNAcylation (Figure 7A). The structure of GLT-1 was predicted, and Thr-551 was labeled with a red sphere (Figure 7B). Then, co-IP was performed to test the interaction between OGT and GLT-1 in astrocytes. As shown in Supplemental Figure 10A, GLT-1 directly combined with OGT in cultured astrocytes. Subsequently, to examine the O-GlcNAcylation of GLT-1, cultured astrocytes were collected, immunoprecipitated with GLT-1, and blotted with O-GlcNAc and GLT-1 antibodies. The co-IP data showed that GLT-1 was O-GlcNAcylated by OGT and that an OGT inhibitor (OSMI-1) decreased the O-GlcNAcylation of GLT-1 in cultured astrocytes (Figure 7, C and D). Moreover, in the CSDS, the O-GlcNAcylation of GLT-1 was specifically increased in the mPFC in the Sus subgroup, but not in the control or Res mice (Figure 7, E and F), without influencing the expression of GLT-1 (Supplemental Figure 10, B and C), suggesting that social stress increases the O-GlcNAcylation of GLT-1 in the mPFC. Furthermore, we employed astrocytic OGT-KO mice to investigate whether the O-GlcNAcylation of GLT-1 was regulated by astrocytic OGT in the mPFC. Tissue from the mPFC was collected, and the co-IP analysis showed that GLT-1 O-GlcNAcylation was significantly reduced in the mPFC in the astrocyte-specific cKO mice compared with that in the WT controls (Figure 7, G and H), but this reduction did not affect the total expression of GLT-1 (Supplemental Figure 10, D and E) as in our previous proteomic analysis (41). These results indicate that astrocytic OGT regulates the O-GlcNAcylation of GLT-1 in the mPFC.

To investigate the effect of GLT-1 O-GlcNAcylation on glutamate-uptake ability, a lentivirus vector with full-length WT GLT-1 or Thr-551 mutant recombinant plasmid was constructed and transfected into HEK293T cells separately (Supplemental Figure 10F). Then, 200 μM l-glutamate was added, and the glutamate-uptake ability was determined by measuring the glutamate levels in the medium after 4 hours. The concentration of glutamate was reduced by almost 40% after 4 hours in the cells transfected with WT GLT-1 compared with that in the cells transfected with the blank plasmid, while the OGT inhibitor (OSMI-1) treatment was sufficient to increase the glutamate-uptake ability in the cells transfected with the WT GLT-1 plasmid (Figure 7I). Furthermore, the cells transfected with the GLT-1 Thr-551 mutant plasmid showed significantly lower glutamate concentrations than the cells transfected with the WT GLT-1 plasmid, indicating a higher glutamate-uptake ability. However, the OGT inhibitor failed to increase glutamate uptake in the mutant GLT-1–transfected cells (Figure 7I). These results suggest that OGT regulates glutamate-uptake ability through the O-GlcNAcylation of GLT-1 at Thr-551 in vitro.

**Figure 8:**
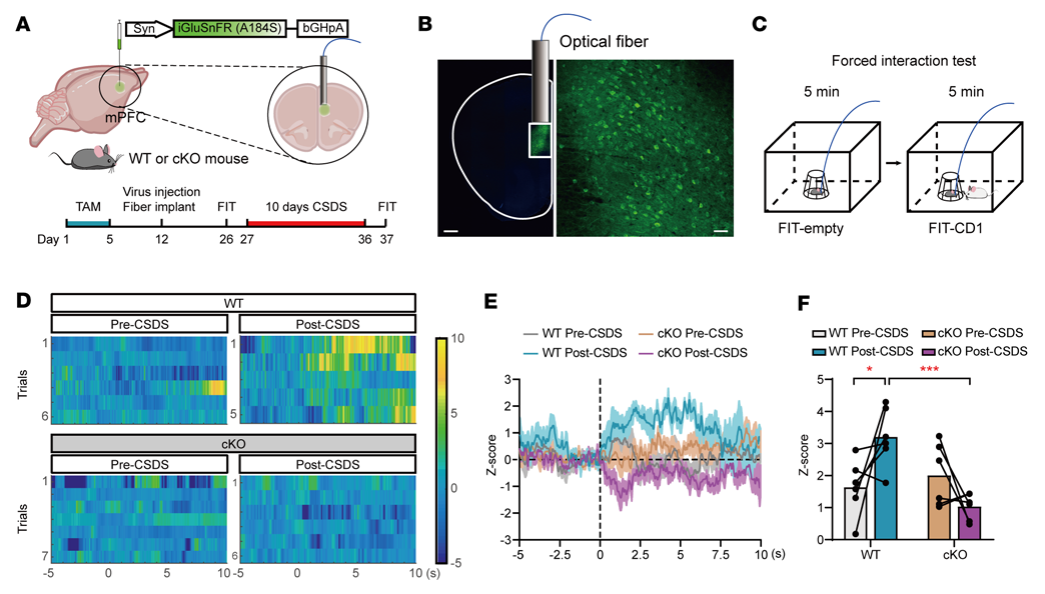
Astrocytic OGT regulates glutamate signaling via the O-GlcNAcylation of GLT-1. (**A** and **B**) Schematic of the AAV vectors engineered to express the glutamate sensor (iGluSnFR A184S) under a *syn* promoter (**A**) and representative images of the injection sites in the mPFC (**B**). Scale bars: 500 μm (right, magnified view of the left image); 50 μm (left). (**C**) Paradigms of the FIT. (**D**) Representative heatmaps of *z* score changes over all trials in single mice. (**E** and **F**) Time course of average iGluSnFR transient *z* scores event locked to SI (**E**) and quantification of the average peak *z* score during SI (**F**). *n* = 6 (WT); *n* = 6 (cKO) mice. Data are represented as mean ± SEM. Two-way ANOVA with Bonferroni’s multiple-comparisons test (**F**). *P < 0.05; ***P < 0.001. See Supplemental Data Set 1 for the statistical details.

To determine whether the O-GlcNAcylation of GLT-1 modulates dynamic changes in glutamate in the mPFC, we employed a glutamate sensor (iGluSnFR, A184S) under a syn promoter, which contained the high-affinity SF-iGluSnFR variant and improved the detection of stimulus-evoked glutamate release (48). AAV-iGluSnFR virus was infused into the mPFC of astrocyte-specific cKO and WT control mice, and fiber optics were implanted above the infected cells (Figure 8, A and B, and Supplemental Figure 11, A and B). First, we performed photometry recordings during the SI test in the WT and cKO mice before and after the CSDS paradigm (Supplemental Figure 11, A and C), which allowed us to record the real-time glutamate change when the mice chose to socially interact with CD1. In the glutamate-signaling analysis, no difference was observed between the WT and cKO mice before CSDS; however, the WT mice exhibited higher maximum glutamate signaling than the cKO mice after 10 days of CSDS (Supplemental Figure 11, D–F). Moreover, the change in the maximal *z* score in each mouse was calculated after the CSDS paradigm, and a correlation analysis was performed between the changed *z* score and SI ratio; a negative correlation was observed between the glutamate-signaling change and the SI ratio (Supplemental Figure 11G). Subsequently, photometry recordings were performed in WT or cKO mice using a forced interaction test (FIT) assay (Figure 8C), which can directly quantify the glutamate responses to an aggressor mouse without the influence of the exploratory or escape behaviors exhibited during the choice SI test (49). Before the CSDS paradigm, the heatmaps and maximum fluorescence change in the astrocyte-specific cKO and littermate control mice exhibited the same glutamate release while interacting with the CD1 aggressor in the FIT. After 10 days of CSDS, the control mice showed a significantly increased time course of glutamate change during CD1 contact. Interestingly, the astrocyte-specific cKO mice demonstrated obviously attenuated glutamate release in the FIT compared with the control mice (Figure 8, D–F). Taken together, these data prove that astrocytic OGT regulates social-stress–induced dynamic changes of glutamate signaling in the mPFC through the O-GlcNAcylation of GLT-1.

### OGT reduction in astrocytes prevents the disruption of glutamatergic synaptic transmission from stress.

**Figure 9:**
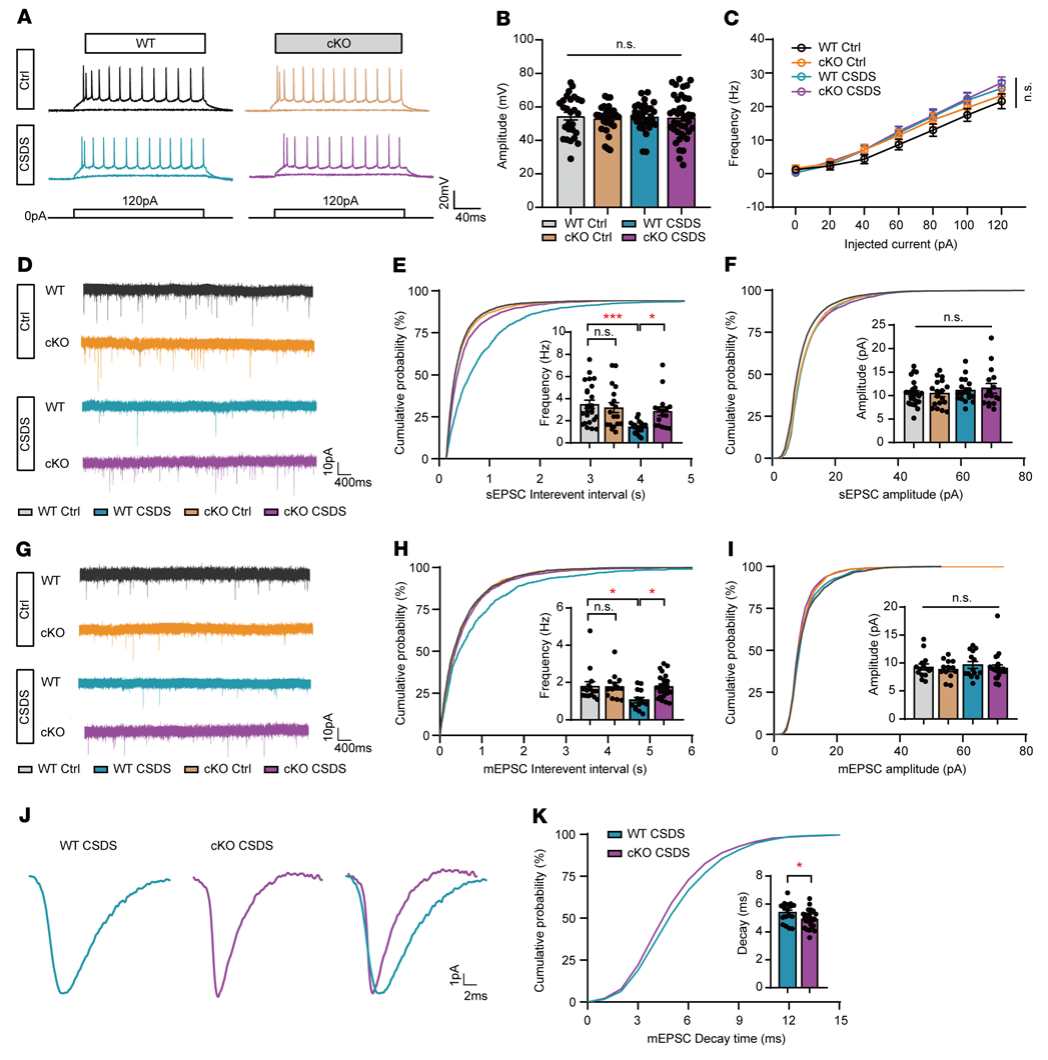
OGT reduction in astrocytes prevents the disruption of glutamatergic synaptic transmission from stress. (**A**) Representative action potential firing traces of whole-cell current clamp recordings from mPFC layers II and III pyramidal neurons of WT and cKO mice with or without the CSDS paradigm. Raw traces show individual voltage responses to a series of 500 ms current pulses from 0 to 120 pA in 20 pA steps. (**B** and **C**) The amplitude of action potentials (**B**) and the evoked spike rates versus current magnitudes (I/O curve) (**C**) of pyramidal neurons from WT and cKO mice with or without the CSDS paradigm. (**D**) Representative traces of sEPSCs recorded from WT and cKO mice with or without the CSDS paradigm. (**E** and **F**) Cumulative distribution and mean sEPSC frequency (**E**) and amplitudes (**F**). *n* = 18–24 cells from 4 individual mice. (**G**) Representative traces of mEPSCs recorded from WT and cKO mice with or without the CSDS paradigm. (**H** and **I**) Cumulative distribution and mean mEPSC frequency (**H**) and amplitudes (**I**). *n* = 13–23 cells from 4 individual mice. (**J**) Representative traces of mean individual mEPSCs from WT and cKO mice after CSDS. (**K**) Cumulative distribution and mean value of mEPSC decay time. *n* = 17 (WT CSDS); *n* = 25 (cKO CSDS) from 4 individual mice. Data are represented as mean ± SEM. Two-sided unpaired *t* test (**K**); 2-way ANOVA with Bonferroni’s multiple-comparisons test (**B**, **C**, **E**, **F**, **H**, and **I**). *P < 0.05; ***P < 0.001. See Supplemental Data Set 1 for the statistical details.

To explore the influence of stress on synaptic transmission, we first employed whole-cell, patch-clamp recording to record the action potentials of pyramidal neurons in layers II and III. The amplitude and number of action potentials triggered by a series of current injections were the same in the 2 groups with or without stress (Figure 9, A–C). Then, we recorded spontaneous excitatory postsynaptic currents (sEPSCs) in the mPFC of astrocyte-specific cKO and WT mice. After 10 days of CSDS, chronic stress reduced the frequency of sEPSCs in the WT mice, while the frequency of sEPSCs in the astrocytic OGT-cKO mice was preserved under stress (Figure 9, D and E). No difference in the amplitude of sEPSCs was observed between the 2 groups (Figure 9, D and F). Furthermore, to examine the influence of basal glutamatergic synaptic transmission in astrocyte-specific cKO and WT mice, we compared the miniature excitatory postsynaptic currents (mEPSCs) of pyramidal neurons in the mPFC. The frequency of mEPSCs was significantly decreased in the WT mice after CSDS. In contrast, the frequency of mEPSCs was maintained in the astrocyte-specific OGT-cKO mice after CSDS and significantly differed from that in the WT mice after stress (Figure 9, G and H). The mEPSC amplitudes in the pyramidal neurons did not differ between the cKO and WT mice (Figure 9, G and I). Additionally, we analyzed the decay time of single mEPSCs, and the data showed that the decay time of mEPSCs was elevated in the WT mice compared with that in the cKO mice after CSDS, indicating longer glutamate clearance in synapses (Figure 9, J and K). These results suggest that the OGT reduction alleviated the disruption of glutamatergic transmission after chronic social stress.

### OGT loss of function in the mPFC preserves neuronal morphology and calcium activity under social stress.

**Figure 10:**
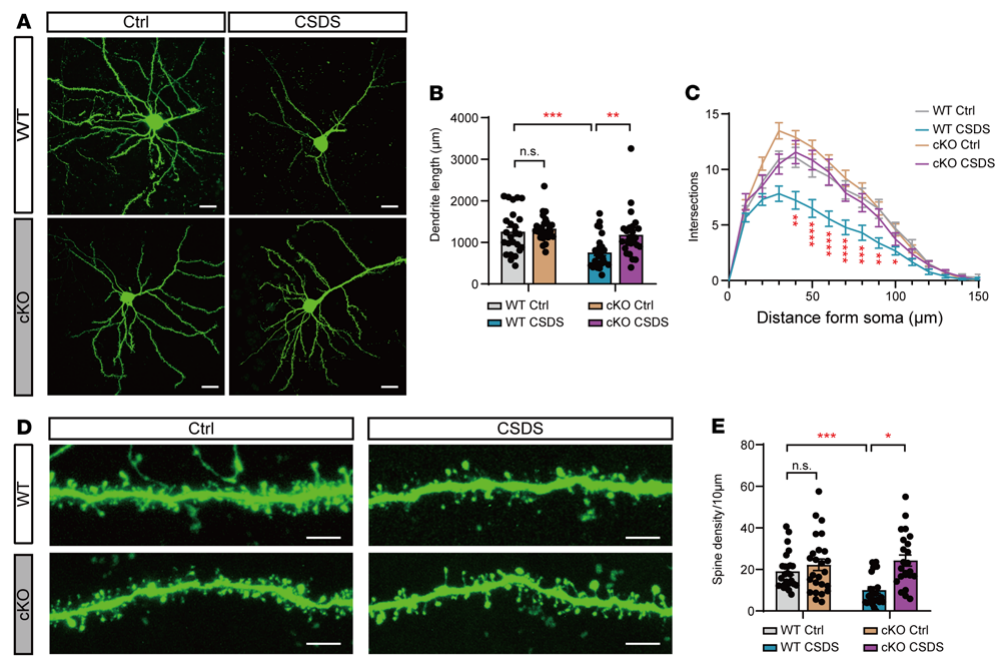
OGT loss of function in the mPFC preserves neuronal morphology under social stress. (**A**) Representative confocal micrographs showing EGFP-expressing signal pyramidal neurons in the mPFC of OGT-cKO mice and WT mice exposed to the CSDS paradigm. Scale bars: 20 μm. (**B**) Total cumulative lengths of basal dendritic processes per EGFP-positive neuron in the mPFC. *n* = 25–27 cells from 6 individual mice. (**C**) Sholl analysis of dendritic arbors of pyramidal neurons from cKO and WT mice. *n* = 25–27 cells from 6 individual mice. (**D**) Representative confocal images showing the dendritic spines of EGFP-positive pyramidal neurons in the mPFC of OGT-cKO and WT mice suffering from the CSDS paradigm. Scale bars: 5 μm. (**E**) Quantitative analysis of dendritic spine density. *n* = 23–26 dendrite segments from 6 individual mice. Data are represented as mean ± SEM. Two-way ANOVA with Bonferroni’s multiple-comparisons test (**B**, **C**, **E**). *P < 0.05; **P < 0.01; ***P < 0.001; ****P < 0.0001. See Supplemental Data Set 1 for the statistical details.

Chronic stress or abnormally high levels of extracellular glutamate cause dendritic atrophy and spine loss (50). To investigate the roles of astrocytic OGT in dendritic arborization and spine density under stress, we sparsely labeled pyramidal neurons in the mPFC of astrocyte-specific cKO and WT mice with a viral cocktail (1:1) of AAV-CaMKIIα-FLP and AAV-nEf1α-FDIO-EYFP. The morphometric analysis showed no obvious difference in the total basal dendrite length or dendritic complexity between the astrocyte-specific cKO and WT mice without social stress (Figure 10, A–C). After 10 days of CSDS, the WT mice exhibited significantly reduced total dendrite length and dendritic complexity compared with the WT mice without CSDS (Figure 10, A–C). In contrast, the total dendrite length and complexity of dendrites in the astrocyte-specific cKO mice were intact after CSDS (Figure 10, A–C). Further analyses of the dendritic spine density revealed no differences in the spine density between the astrocyte-specific cKO mice and littermate controls without social stress (Figure 10, D and E). However, spine density was significantly decreased in the pyramidal neurons of the WT mice after CSDS compared with that in the control mice. In contrast, spine density in the OGT-cKO mice did not change after CSDS and was significantly higher than that of the WT mice under social stress (Figure 10, D and E). These results suggest that astrocytic OGT loss of function reduces dendritic and spine loss in pyramidal neurons under social stress.

**Figure 11:**
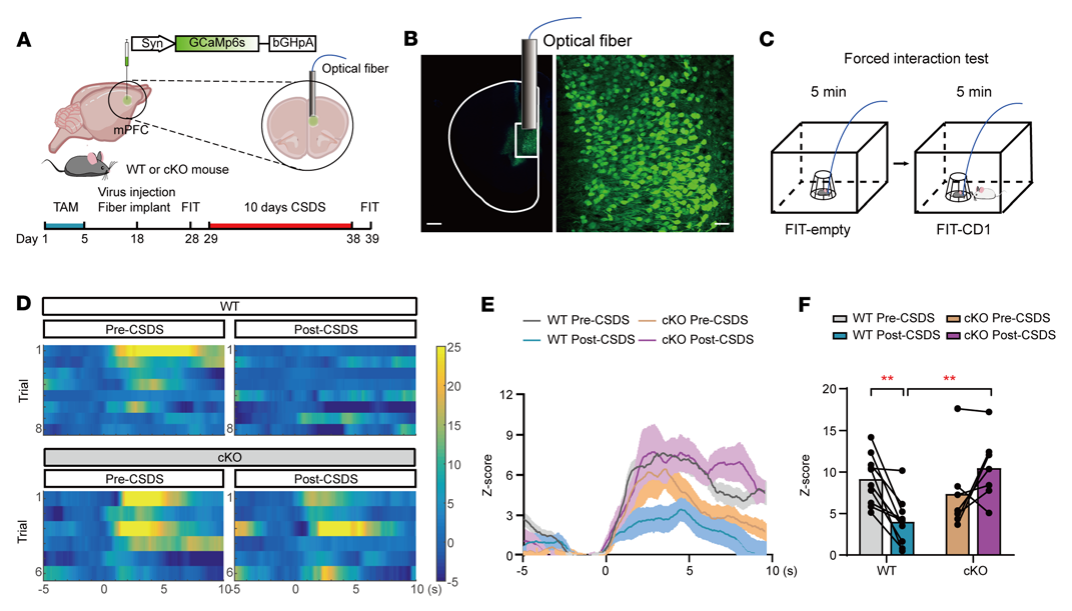
Deletion of astrocytic OGT in the mPFC protects neuronal calcium activity from stress. (**A**) Schematic of the AAV vectors engineered to express a calcium sensor (GCaMp6s) under a syn promoter. (**B** and **C**) Representative images of the injection sites in the mPFC and paradigms of the FIT. Scale bars: 500 μm (right, magnified view of the left image); 50 μm (left). (**D**) Representative heatmaps of *z* score Ca^2+^ signal changes over all trials from single mice. (**E** and **F**) Time course of average GCaMp6s transient *z* scores event-locked to SI (**E**) and the quantification of the average peak *z* score during SI (**F**). *n* = 10 (WT); *n* = 7 (cKO) mice. Data are represented as mean ± SEM. Two-way ANOVA with Bonferroni’s multiple comparisons test (**F**). **P < 0.01. See Supplemental Data Set 1 for statistical details.

Dendritic and spine loss tightly correlate with neuronal function deficits in the mPFC, which are also associated with depression (51). To determine whether astrocytic OGT is involved in regulating neuronal activity in vivo under stress, a Ca^2+^ indicator, GCaMP6s, under a *syn* promoter was applied to investigate calcium activity in the mPFC, and fiber photometry was used to record Ca^2+^ signals in mPFC neurons of WT or OGT-cKO mice during FIT (Figure 11, A–C). Before the CSDS paradigm, the heatmaps and maximum fluorescence changes (*z* scores) showed that the astrocyte-specific cKO and WT mice exhibited the same stimulus-evoked intracellular Ca^2+^ elevations while interacting with CD1 aggressors in the FIT. After 10 days of CSDS, the data showed that the time course of GCaMP6s fluorescence in the WT mice was significantly attenuated in the FIT. In contrast, Ca^2+^ activity was obviously stronger in the OGT-cKO mice during CD1 contact than in the WT mice (Figure 11, D–F), suggesting that astrocytic OGT deletion reverses the neuronal calcium activity deficits in the mPFC caused by chronic stress.

## Discussion

Glucose is the most important energy source in the brain (52). Multiple studies involving depressed humans and animal studies implicate abnormalities in glucose metabolism in the prefrontal cortex (3–5). Most glucose in the brain is used to generate ATP, which also acts as a widespread cell-to-cell signaling molecule. Our previous study showed that astrocytic ATP regulates depressive-like behaviors (27). In addition, some glucose is converted to UDP-GlcNAc through the HBP, which provides GlcNAc for protein O-GlcNAcylation (32). More than 4,000 proteins in all major cell compartments, including the membrane, cytoplasm, mitochondria, and nucleus, have been identified as being O-GlcNAcylated (8, 53). In our previous study, we deleted *Ogt* in astrocytes and found alterations in metabolic processes, transferase activity, and biosynthetic processes (41), but the function of OGT in astrocytes remained unknown. Moreover, O-GlcNAcylation is highly dynamic and reversible and is involved in energy metabolism and stress responses. Fasting leads to a strong reduction in O-GlcNAc in the brain, and OGT in neurons in the paraventricular nucleus of the hypothalamus (PVN) regulates feeding behaviors by regulating excitatory synapse function (54). Stress is an important precursor for depression (17, 18). In the placenta, OGT, as a biomarker of maternal stress, plays a key role in long-term metabolism and disruption of the hypothalamic-pituitary-adrenal (HPA) axis (21). Prenatal stress is associated with sex-specific depressive-like behaviors through placental OGT-related mitochondrial motility (9). A reduction in placental OGT promotes a stress-susceptible phenotype in offspring (21). However, the central role of OGT in stress and mood disorders is still unclear. In the present study, we found that astrocytic OGT in the mPFC was increased in mice with depression (Figure 1). Then, astrocyte-specific cKO mouse lines were generated by crossing the floxed OGT allele with *Fgfr3-iCreER^T2^* transgenic lines, which can label approximately 90% of all protoplasmic and fibrous astrocytes in the adult mouse brain after TAM administration (55, 56). The OGT deletion in astrocytes produced antidepressant effects under stress (Figure 2). However, Fgfr3 is expressed not only in astrocytes, but also neural stem cells in the postnatal SVZ and spinal cord (55, 57). Combined with the specific change of astrocytic OGT in the mPFC, the gABC1D-iCre virus, which preferentially targets astrocytes along with the human GFAP (gfaABC1D) promoter (28), was injected into the mPFC. Mice receiving the gABC1D-iCre virus in the mPFC also exhibited antidepressant-like effects after CSDS (Figure 4), but administration of the virus did not have the same effect in other depression-related brain regions, such as the ventral Stri (Supplemental Figure 7). Moreover, astrocyte-specific gain and loss of OGT in the mPFC bidirectionally regulated depressive-like behaviors in male mice (Figure 4 and Figure 5), which can exclude the influence of neural stem cells in the postnatal SVZ and spinal cord. Our findings indicate that OGT in astrocytes acts as a stress sensor and modulates depression-related behaviors.

Moreover, astrocytes can wrap most excitatory synapses and regulate glutamate uptake by glutamate transporters, which is sufficient for maintaining low extracellular glutamate concentrations by degrading glutamate into glutamine (58, 59). Abnormal glutamate has been implicated in the pathophysiology of depression (60). Postmortem brain analyses of MDD patients and animal model studies show that the concentration of glutamate is significantly increased in the PFC of brains (61, 62), although there have also been reports demonstrating decreased glutamate levels in the brains of adults with MDD (63). However, the plasma levels of glutamate in patients with MDD have been reported to be higher than those in healthy control participants (64). Five weeks of treatment with antidepressants significantly decreased the levels of glutamate in sera (65). GLT-1, the major glutamate transporter in astrocytes, is responsible for approximately 95% of the total glutamate activity in the mature brain (66). The deletion of GLT-1 in mice reduced glutamate uptake and induced depressive-like behaviors (58, 67). By combining O-GlcNAcylation proteomics and co-IP, we confirmed, for what we believe is the first time, that GLT-1 was O-GlcNAcylated by OGT in astrocytes (Figure 4 and Figure 5). Following CSDS, the dynamic change in glutamate signaling was significantly increased in the control mice in the FIT, which is similar to what occurred in previous reports (61, 64). In contrast, the deletion of OGT decreased GLT-1 O-GlcNAcylation and enhanced glutamate uptake under stress (Figure 7 and Figure 8) without affecting the expression of GLT-1 in the mPFC, which is consistent with our previous study (41).

In the mPFC, neurons communicate with each other and serve as a central hub in the emotional circuits of the brain by synapses. Previous studies and our patch-clamp studies showed that the frequencies of sEPSCs and mEPSCs were decreased in mPFC pyramidal neurons under stress (Figure 9) (50, 68), indicating a reduction in presynaptic transmitter release or decreased synaptic connectivity. Glutamate is believed to lack the ability to cross the blood-brain barrier, indicating that glutamate needs to be continuously replenished (58). The decreased glutamate-uptake ability caused by GLT-1 O-GlcNAcylation resulted in a disruption of glutamate-glutamine cycles in astrocytes, which may contribute to the reduction in glutamate release in the presynapse. Interestingly, GLT-1 knockdown in the mPFC also resulted in a decreased frequency of sEPSCs and mEPSCs (69). The astrocyte OGT deletion prevented the disruption of glutamatergic transmission in mPFC pyramidal neurons after CSDS through the O-GlcNAcylation of GLT-1 (Figure 9).

Furthermore, deficits in glutamate reuptake result in increased extracellular glutamate levels and evoke neuronal dysfunction, damage, or death through glutamate excitotoxicity (70). Studies in both depressed patients and animals have demonstrated obvious reductions in the density of neurons, dendritic arborization, and spine loss in the mPFC (25, 68, 71, 72). Here, we found that social stress induced dendritic and spine loss in pyramidal neurons in WT mice, but not in astrocytic OGT-deletion mice. The protection of dendrites in the mPFC prevented neuronal Ca^2+^ activity deficits and resulted in antidepressant effects (Figure 10 and Figure 11).

In this study, we identified a stress and metabolic sensor, OGT, that was sufficient to bidirectionally regulate susceptibility to stress and modulate glutamatergic transmission through the O-GlcNAcylation of GLT-1, resulting in antidepressant effects. However, several interesting questions remain unanswered. Although we show that social stress increases the OGT levels in mPFC astrocytes, but not in neurons, the molecular mechanisms controlling this differential change require further research. Meanwhile, in postmortem brain studies, we notice that the change of *Ogt* was not constant in different data sets of MDD patients (35, 36, 73). In these data sets, all reports are based on a relatively small sample size. Besides, many aspects have been extensively addressed as confounding factors, such as age, sex, postmortem interval (PMI), pH of the brain, dissection techniques, RNA extraction, agonal state of the subjects, and medications taken. In addition, the different manner of death of the controls — for example, the brain samples used in GSE80655 were from sudden deaths as controls (35), whereas all of the control brain samples in GSE102556 were from suicide (73) — may affect gene transcription in postmortem brain studies (74). Given the limited sample size, limitations in postmortem tissue availability and quality, and the heterogeneity and complexity of MDD, further investigations using larger samples are needed. In addition, due to the complication of O-GlcNAcylation and the limitation of O-GlcNAcylation proteomics, we may have missed some O-GlcNAcylated sites in the mPFC. The *Ogt* gene localizes at *Xq13*, and the change in *Ogt* mRNA in blood also exhibited sex differences. In our study, we adopted male mice for the depression models; therefore, the influence of *Ogt* in females requires additional investigation.

Our findings uncovered the role of OGT deletion in antidepressant-like effects by protecting mPFC pyramidal neurons from glutamate-transmission deficits under social stress through the O-GlcNAcylation of GLT-1. These results establish a causal relationship between O-GlcNAcylation and mood disorders, and the O-GlcNAcylation site of GLT-1 may provide new insights into stress-induced depression targeting neuron-glia interactions.

## Methods

The procedures used for human subjects and animals, virus injection, optical fiber and cannula implantation, fiber photometry, cell culture, Western blot and Simple Western analysis, FACS, co-IP, immunofluorescence staining, real-time qPCR, plasmid construction and cell transfection, evaluation of glutamate-uptake ability, behavioral studies, ex vivo electrophysiological recordings, proteomics analysis of O-GlcNAcylation, and postmortem brain tissue bioinformatics analysis are detailed in Supplemental Methods. See the supplemental material for full, uncut gels.

### Statistics.

All experiments and data analyses were conducted in a blinded manner. The numbers of replicates (*n*) are indicated in the figure legends. Data are represented as mean ± SEM. Statistical comparisons were performed using SPSS, version 20.0, and Prism, version 7.0, with appropriate inferential methods, as indicated in the figure legends. Normally distributed data were tested by 2-sided unpaired *t* test for 2-group comparisons and 1- and 2-way ANOVA followed by Bonferroni’s test for multiple comparisons. Nonnormally distributed data were analyzed by Mann-Whitney *U* tests for 2-group comparisons and Kruskal-Wallis test and Dunn’s multiple-comparisons test for more than 2 groups. Statistical significance was set at *P* < 0.05.

### Study approval.

All animal experiments were conducted in accordance with the Chinese Council on Animal Care Guidelines, and ethics approval was obtained from the Research Ethics Board at Southern Medical University. The use of peripheral blood from MDD patients was approved by the Ethics Committee of Guangdong 999 Brain Hospital (no. 2021-01-087). This study was performed in accordance with the Declaration of Helsinki. Informed consent was obtained from all individual participants included in the study. The authors are responsible for the accuracy of the statements provided in the manuscript.

## Author contributions

XC and JF designed the study and wrote the paper. XC, JF, and FG analyzed the data. JF performed most of the experiments. JF, FG, RM, and JWM performed the behavioral experiments with the help of YYF and the stereotactic injection and in vivo recordings with the help of CLL and JR. JF, FG, and QLZ performed the Western blot and co-IP analyses. JWM performed the FACS and Simple Western analyses. JR and SJL were responsible for cell culture. JF and FG performed the patch-clamp experiments. YLW and TTG collected blood from MDD patients and healthy controls with the help of LYC. XJK and QLZ performed qPCR analysis. JML and RM carried out genotyping. LYC, FG, and CZ analyzed the bioinformatics data. TMG reviewed and edited the manuscript. XC supervised all phases of the project.

## Supplementary Material

Supplemental data

Supplemental data set 1

Supplemental data set 2

Supplemental tables 1-3

## Version 1 02/09/2023

In-Press Preview

## Version 2 04/03/2023

Electronic publication
