# Supplementary material for: O-GlcNAc transferase in astrocytes modulates depression-related stress susceptibility through glutamatergic synaptic transmission
Source: J Clin Invest. 2023 Apr 3;133(7):e160016. doi: 10.1172/JCI160016 (PMC10065078; doi:10.1172/JCI160016)

Supplemental Materials

Methods

Human subjects

The participants were required to meet the following criteria: (1) meet the diagnostic criteria for MDD using the Structured Clinical Interview for DSM-IV Axis I Disorders; (2) a detailed assessment of previous psychiatric symptoms and treatment and an assessment of the severity of depression using the 24-item Hamilton Depression Rating Scale (HAMD-24) performed by two psychiatrists (Y-L Wen and T-T Gu); (3) first-episode depression patients without any medications or drug-free for longer than 3 weeks; and (4) aged between 18 and 50 years. The exclusion criteria for the MDD group included the following: (1) other major psychiatric disorders or neurodegenerative disease history; (2) head trauma, substance abuse, or loss of consciousness; and (3) a history of any inflammatory diseases, autoimmune diseases, or metabolic diseases. The healthy control subjects were required to have a HAMD-24 score ≤ 8 and Symptom Check List 90 score ≤ 90. The exclusion criteria included a history of neuropsychiatric disorders, head trauma, drug abuse, or insobriety.

The use of peripheral blood from MDD patients was approved by the Ethics Committee of Guangdong 999 Brain Hospital, Guangzhou, Guangdong (NO. 2021-01-087). The healthy control participants were recruited through a local community posting. Written informed consent was required from all participants, and the study was performed in accordance with the Declaration of Helsinki (ethical principles for medical research involving human subjects).

Animals

Four to five mice were socially housed and maintained under standard housing conditions on corn cob litter in a temperature- (23 ± 1℃) and humidity- (40%) controlled animal room under a 12 h light/dark cycle (lights were on from 07:00 to 19:00 every day) with free access to food and water.

C57BL/6J mice (aged 8-12 weeks) were obtained from the Southern Medical University Animal Center (Guangzhou, China). $OGT^{flox/flox}$ and $CaMK\,II\,\alpha\text{-}creER^{T2}$ mice were purchased from Jackson Laboratories (Stock No. 004860 and No. 012362, USA). $Fgfr3\text{-}iCreER^{T2}$ mice (C57BL/6J background) were generously provided by William D Richardson (University College London, London, UK).

$Fgfr3\text{-}iCreER^{T2}; OGT^{flox/Y}$ mice were generated by crossing $OGT^{flox/flox}$ mice with $Fgfr3\text{-}iCreER^{T2}$ mice. $CaMK\,II\,\alpha\text{-}creER^{T2}; OGT^{flox/Y}$ mice were generated by crossing $OGT^{flox/flox}$ mice with $CaMK\,II\,\alpha\text{-}creER^{T2}$ mice. To excise the *loxp* sites by *Cre* recombination, 2-month-old male mice were injected with Tamoxifen (TAM) (Sigma, USA) once a day (intraperitoneally (i.p.), 100 mg kg^{-1} of body weight) for 5 consecutive days. TAM was dissolved in corn oil (Sigma, USA) at a final concentration of 10 mg ml^{-1}. Littermate $OGT^{flox/Y}$ mice injected with TAM were used as controls.

Only male mice (8-13 weeks old) with a normal appearance and weight were used for all tests. All behavioral tests were conducted by experimenters who were blinded to the experimental groups.

Virus injection

Two-month-old male mice were anesthetized with isoflurane and fixed in a stereotactic frame (RWD, China) before surgery. The virus was stereotaxically

injected into the bilateral mPFC (AP: −1.75; ML: ±0.3; DV: −2.5; mm, where AP denotes anteroposterior from bregma, ML denotes mediolateral from the midline and DV denotes dorsoventral from the brain surface). A volume of 0.6-0.8 μl virus (depending on the expression strength and viral titer) was injected using a 5 μl Hamilton (USA) microsyringe fitted with a 33-gauge needle and a microsyringe pump (Stoelting, USA) at a rate of 0.1 μl min^{-1}. The syringe was not removed until 5 min after the end of the infusion to allow the diffusion of the virus.

For the specific knockdown of astrocytic OGT, the virus purchased from Taitool Bioscience containing (China) iCre under a gfaABC1D promoter (AAV2/9-gfaABC1D- eGFP-iCre-SV40pA) or control virus (AAV2/9-gfaABC1D-eGFP-bGHpA) was bilaterally injected into the mPFC of $OGT^{flox/Y}$ mice. To overexpress OGT in astrocytes, the virus (AAV2/8-CMV-DIO-OGT-3×Flag-WPRE-PA) or control virus (AAV2/8-CMV-DIO-3×Flag-WPRE-PA) (BrainVTA, China) was bilaterally injected into the mPFC of *Fgfr3-iCreER*^{T2} mice, and TAM was administered 7 days after the virus injection.

For the sparse labeling of pyramidal neurons, *Fgfr3-iCreER*^{T2}; $OGT^{flox/Y}$ cKO and control mice were injected with a total 0.4 μl viral cocktail (1:1) of AAV2/9-CaMK Ⅱ α-FLP-WPRE-PA and AAV2/9-nEf1α-FDIO-EYFP-WPRE-PA (BrainVTA, China) in the mPFC (AP: −1.75; ML: ±0.3; DV: −2.5; mm). Two weeks after the final injection, both the cKO and WT mice were subjected to 10 days of CSDS or non-CSDS control, and the mice were euthanized. Brain sections were collected for confocal imaging. The basal dendritic complexity and spine density of pyramidal neurons were analyzed with Imaris 8.0 by investigators who were blinded to the experimental group.

Cannula implantation

For the pharmacological experiments, a bilateral cannula (RWD, China) was implanted above the mPFC core (AP: −1.75; ML: ±0.35; DV: −2.5; mm) of 2-month-old male C57BL/6J mice for the infusion of the OGT antagonist OSMI-1 (50 μM in 0.5 μl, Sigma, USA). The cannula and screws were held in place with dental cement. A stainless-steel obturator was inserted into each guide cannula to prevent blockage. Only mice with the correct locations of the cannula were used for further analysis.

Fiber photometry

A total of 0.3-0.4 μl of AAV2/9-iGluSnFR(A184S)-WPRE-hGHpA or AAV2/9-hSyn-GCaMp6s-WPRE-hGHpA (BrainVTA, China) was infused into the mPFC at the same stereotactic coordinates previously noted, and a fiberoptic implant was advanced and secured at the same location. After allowing 2 weeks for viral expression, the mice were first habituated to the fiber photometry apparatus for 30 min and then tested on a subsequent day. Continuous video and fiber photometry acquisition were recorded during the SI test or FIT with an aggressive CD1 mouse. To record the fluorescence signals, a laser beam from a laser tube (488 nm) was reflected by a dichroic mirror focused by a 10× (NA of 0.3) lens and coupled to an optical commutator. The fluorescence was bandpass filtered (Thorlabs, USA) and collected by a photomultiplier tube (Hamamatsu, Japan). An amplifier (Hamamatsu, Japan) was used to convert the photomultiplier tube current output to voltage signals, which were further filtered through a low-pass filter (40 Hz cutoff; Brownlee 440). To minimize photobleaching, the laser power at the fiber tip was adjusted to 30 μW.

Signal processing was performed with MATLAB (MathWorks, USA). The z-score of a population of astrocytes was calculated using the following formula: z-score = $(F_{Signal} - F_{Basal})/STD(F_{Basal})$. The videos were manually analyzed by a genotype-blinded observer, who determined the time of interaction (WT or cKO mouse-to-CD1 contact in SI or CD1-to-cKO or WT mouse contact in FIT). Peristimulus time histograms were constructed by calculating the average of 15 s nonoverlapping epochs of fluorescence consisting of 5 s before and 10 s after the contact time, which was defined as time=0. The peak z-scored fluorescence was determined for each peristimulus time histogram as the maximal z-score value between 0 and 10 s.

Cell culture

Tissues isolated from the hippocampus or cerebral cortex of postnatal day 1 mice were washed with ice-cold PBS and transferred to a 50 ml Falcon tube containing 0.5 ml PBS for dissociation using a pair of sterile operating scissors. Then, the tissues were incubated with 0.25% trypsin (Thermo Fisher Scientific, USA) in 0.5 mM EDTA at 37°C for 10 min. Culture medium was added to inhibit trypsin, and the cell suspension was transferred into 15 ml tubes and centrifuged at 9000× g for 6 min. The pellet was resuspended in 10 ml of culture medium (Thermo Fisher Scientific, USA) (for neurons, Start Medium: Neurobasal Medium-A + 1% B27+ 1% Glutamax + Glutamate 25 μM; Culture Medium: Neurobasal Medium-A + 1% B27 10 ml + 1% Glutamax; for astrocytes, DMEM F12 + 10% FBS). The cells were placed in a culture flask at a density of 5×10^{6} cells per 5 ml and incubated in a humidified incubator containing 5% CO_2 air at 37°C.

Western blot analysis

Cultured cells and mouse brain tissues (including the mPFC, Stri, NAc, Hip, Amy, and DRN) were homogenized in lysis buffer (RIPA, Thermo Fisher Scientific, USA) in the presence of protease inhibitor (PMSF, Thermo Fisher Scientific, USA), phosphatase inhibitor cocktails (Sigma, USA), and an O-GlcNAcylation inhibitor (PUGNAc) (Sigma, USA) on ice for 30 min and centrifuged at 13,000 rpm for 20 min at 4°C. The protein samples (30-60 μg) were resolved by 10% sodium dodecyl sulfate–polyacrylamide gel electrophoresis (SDS–PAGE) gels and subsequently immunoblotted onto polyvinylidene difluoride (PVDF) membranes (Millipore, Germany) in ice-cold buffer (25 mM Tris HCl, 192 mM glycine, and 20% methanol) by electrotransfer for 2 h. The membranes were incubated with the indicated antibodies at 4°C overnight: anti-OGT (Abcam, ab96718, 1:1000, England); anti O-GlcNAc (PTM Biolab, PTM-952, 1:1000, China) ; and anti-GLT-1 (Abcam, ab205247, 1:1000, England). The samples were incubated with dye-680-goat-anti-rabbit (Li-cor, 925-68071, 1:5000, USA) and/or dye-800-goat-anti-mouse (Li-cor, 926-32210, 1:5000, USA) secondary antibodies at room temperature for 1 h. The membranes were then scanned on an Odyssey infrared imaging system (Li-cor Biosciences, USA), and images were acquired and analyzed according to the manufacturer's instructions.

Fluorescent-activated cell sorting

The mice were anesthetized with isoflurane and perfused with ice-cold sterile PBS. Their brains were dissected, coarsely chopped and incubated in 5 ml EBSS solution containing 2 mg ml^{-1} papain (Sigma, USA), 1 mg ml^{-1} L-cysteine (Sigma, USA), 0.5 mg ml^{-1} EDTA (Sigma, USA) and 100 μg ml^{-1} DNase I (Sigma, USA) for 30 min at

37°C. In the middle of the incubation, the brain tissue was dissociated with an 800-μm pipette approximately 15 times every 15 min. For the enrichment of astrocytes, the cell pellet was resuspended in 5 ml of 26% Percoll (Sigma, USA) solution and centrifuged at 900 g and 4°C for 15 min. Then, the cell pellet was resuspended, passed through a 40-μm mesh, and subjected to antibody labeling and a FACS analysis.

Cell surface markers were stained for 30 min at 4°C with FcR blocking reagent (Miltenyi, USA) and an anti-ACSA-2-PE (Miltenyi, USA) monoclonal antibody. Then, the cells were analyzed and acquired using a MoFlo XDP flow cytometer. For the OGT-positive astrocyte analysis, OGT was detected in fixed and permeabilized cells with anti-OGT (Abcam, England). The data were collected by an Accuri flow cytometer system (BD LSRFortessa X-20, USA) and analyzed with FlowJo software.

Simple Western

Astrocytes were lysed in RIPA buffer with 1% phenylmethylsulfonyl fluoride (PMSF). The samples were then mixed with Simple Western sample buffer and standards to a final concentration of 1 $\mu g\ \mu l^{-1}$, reduced and denatured. For the protein detection, we used Simple Western (Protein Simple, USA). The target proteins were identified with a primary antibody and subsequent immunodetection using a horseradish peroxidase (HRP)-conjugated secondary antibody and chemiluminescent substrate.

Coimmunoprecipitation

Brain tissue or cell lysates were precipitated by adding anti-GLT-1 antibodies (Abcam, USA, 1:40). After incubation at 4°C overnight, the complexes were

precipitated with 50 μl of protein A-Sepharose beads with gentle agitation at 4°C for 4 h, and nonspecific IgG was used as a negative control. The beads were washed, and the immunoprecipitated protein complex was loaded onto a 10% SDS–PAGE gel and processed for a Western blot analysis.

Immunofluorescence staining

Cultured cells were seeded on coverslips to the appropriate density. The cells were fixed in 4% paraformaldehyde for 15 min and permeabilized in 0.5% Triton X-100 solution for 5 min at room temperature. The animals were perfused transcardially with saline, followed by 4% PFA in 0.1 M PBS, pH 7.4. The brains were removed, postfixed overnight in 4% PFA at 4°C and transferred to 30% sucrose in 0.1 M PBS, pH 7.4. Coronal sections (40 μm) were cut using a cryostat (Leica CM3000, Germany). After washing with PBS 3 times, the sections were incubated in blocking buffer containing 5% normal goat serum in 0.2% Triton X-100/PBS (PBST) for 1 h at room temperature and then with primary antibodies in blocking buffer overnight at 4°C. Then, the samples were washed and incubated with a secondary antibody for 1 h at room temperature. The nuclei were counterstained with DAPI. The coverslips were mounted onto glass slides with anti-fade solution and visualized under a Nikon fluorescence microscope.

RNA preparation for real-time quantitative PCR

The total RNA was extracted from whole blood using TRIzol (Thermo Fisher Scientific, USA) according to the manufacturer's instructions, and the RNA samples were stored at -80°C. One microgram of total RNA was used to synthesize cDNA by a Transcriptor First Strand cDNA Synthesis kit (TAKARA, Japan). Quantitative PCR

was performed using TB Green® Premix Ex Taq™ II (TAKARA, Japan) on a LightCycler 96 Real-Time System (Roche, Switzerland). The following primers were used in the qPCR analysis: *Ogt*: 5'-AAGAGGCACGCATTTTTGAC-3', Reverse 5'-ATGGGGTTGCAGTTCGATAG-3'; *β-Actin*: 5'-CCACCATGTACCCTGGCATT-3', Reverse 5'-GCAGGAGTATGACGAGTCCG-3'. Each reaction was performed in triplicate. A fragment of Actin was amplified as the internal control. The differences in gene expression were calculated by the $2^{-\Delta\Delta CT}$ method and are presented as the fold change.

Plasmid construction and cell transfection

Flag-EGFP-tagged full-length GLT-1 plasmids and Thr-551 mutation plasmids were constructed (BrainVTA, China). H293T cells were grown to 70% confluence and transfected using TransIT-X2 (Mirus Bio, USA) according to the manufacturer's protocol. The cells were transfected for 24 h at 37 ℃.

Evaluation of glutamate uptake ability

H293T cells (American type culture collection, ATCC, USA) were transfected with blank, WT GLT-1 or Mut. The GLT plasmid was treated with or without an OGT inhibitor (50 μM) for 4 h. The cells in each group were treated with 200 μM L-glutamate (Sigma, USA) and incubated for 4 h at 37 ℃. At each time point, the concentration of glutamate in the medium was measured with a glutamate assay kit (Abcam, England) according to the manufacturer's protocol, and the glutamate uptake ability was calculated as follows: (concentration $_{4\,h}$ − concentration 0 h)/concentration $_{0\,h}$ ×100%.

Behavioral studies

For all behavioral assays, mice were transported to the testing room, where they were habituated at least 1 d before testing. During the testing session, the behavior of the animals was recorded using a video tracking system. Dim light (~20 lux) was used in the room to minimize the anxiety of the animals. The experimenter was blinded to the group identity during the experiment and quantitative analyses.

Chronic social defeat stress

According to the CSDS protocol, an adult male C57BL6/J mouse (intruder) was exposed to a different aggressor mouse (retired male breeder CD1 mouse) for 10 min each day for 10 continuous days. After 10 min of physical interaction, the test mouse and the aggressor were separated by a perforated translucent plastic divider. The test mouse was exposed to chronic stress in the form of a threat for the next 24 h. The control (nondefeated) mice were housed in equivalent cages but with members of the same strain. The mice that were placed with the controls were changed daily. During the treatment, all mice were housed individually following the CSDS protocol, and avoidance behavior was tested 24 h following the treatment as described below.

Subthreshold social defeat stress

This procedure was identical to the normal chronic social defeat stress procedure, except for the procedure lasted for three consecutive days.

Social interaction test

Social avoidance behavior was measured using a two-stage social interaction test. The mice were placed in a novel cage (44×44×44 cm^3) containing an empty metal cage (9.5×9.5×8 cm^3), and their movement was tracked for 2.5 min in the absence of the aggressor. Their movement was then followed for 2.5 min in the presence of the caged aggressor. The duration of time that the mice spent in the interaction zone (an

8-cm region flanking the cage) and other measures were obtained using EthoVision 11.0 software (Noldus, Netherlands). The apparatus was cleaned with a solution of 70% ethanol in water to remove olfactory cues following each trial, and all behavioral tests were conducted in the dark. An interaction ratio (time spent in the interaction zone in the presence versus the absence of a target mouse) of 1 was set as a cutoff. Mice with scores <1 were considered susceptible, and those with scores ≥ 1 were considered unsusceptible.

Forced interaction test

A cKO or WT mouse was placed in an 8 cm × 15 cm Plexiglas cylinder. Following a 5 min recording period during which fluorescence signals were recorded, a CD1 aggressor mouse was introduced in the cage outside of the cylinder (18 cm high walls surrounded the outer cage to prevent escape, and a lid was placed over the inner chamber to prevent the aggressor from climbing in). Fluorescence signals were then recorded for an additional 5 min. All animals were subjected to FIT both before and after exposure to CSDS.

Forced swimming test

Mice were placed in a container filled with water that eventually resulted in immobility, reflecting behavioral despair. The FST was performed in a clear glass cylinder (height 45 cm, diameter 19 cm) filled to 23 cm with water (23±1°C). The test lasted for 6 min. The duration of immobility was recorded during the final 4 min by EthoVision 11.0 software (Noldus, Netherlands).

Open field test

To explore locomotion and spontaneous activity, we characterized the behavior of mice as they freely explored an open-field plastic chamber ($40 \times 40 \times 30$ cm^3, width×length ×height). The mice were placed in this arena for 5 min, and the total

distance traveled and time spent in the center region (20×20 cm^2) were recorded by a VersaMax Animal Activity Monitor system (USA) and analyzed by VersaMax 4.20 software (USA).

Light-dark box test

In the LD, a black Plexiglas box ($40 \times 20 \times 12$ cm^3) was placed on one side of the open field test chamber ($40 \times 40 \times 30$ cm^3), which divided the chamber into two compartments of equal size. Mice were placed in the corner of the light compartment and allowed to explore freely for 5 min. The duration in each area and the first latency and total entries to the dark compartment were detected by a VersaMax Animal Activity Monitor system (USA) and analyzed by VersaMax 4.20 software (USA).

Elevated plus maze

The apparatus consisted of two opposing open arms ($30 \times 5 \times 0.5$ cm^3) and two opposing enclosed arms ($30 \times 5 \times 15$ cm^3) that were connected by a central platform (5×5 cm^2) and positioned 50 cm above the ground. The behavior was tracked for 5 min with an overhead camera and EthoVision 11.0 software (Noldus, Netherlands). The time spent in the open arms and close arms was reported.

Novelty-suppressed feeding test

After 24 h of food deprivation and water available ad libitum, mice were placed in a brightly lit open arena ($50 \times 50 \times 50$ cm^3) containing clean wood chip bedding. A filter paper (8×8 cm^2) was placed in the center of the arena, and one familiar food pellet was placed on the center of the filter paper. The mice were removed from their home cage, placed in a holding cage for 60 min before testing and then placed in a corner of the testing arena. The latency to begin a feeding episode was recorded with a video camera suspended above the arena and saved for further analysis (EthoVision by

Noldus, Netherlands). Immediately after testing, the mice were removed from the arena and placed in their home cage to measure food consumption for 5 min.

Rotarod test

Mice were placed on a stationary rotarod (AccuRotor Rota Rod Tall Unit, 63 cm fall height, 30 mm diameter rotating dowel). The dowel was accelerated to 60 RPM in 5 min, and the latency to fall (in seconds) was recorded. The pretraining procedure was repeated over 3 consecutive trials, the training lasted for 4 days, and each mouse was immediately placed on the dowel while falling off the dowel until the end of the test. The latency to fall was measured as previously described.

T maze

The T maze consisted of three identical arms ($30\times10\times20$ cm^3) connected by a center square area (10×10 cm^2). The two arms that were connected in a line were defined as the goal arms, while the last arm was defined as the start arm. The mice were subjected to two trials per day for 10 consecutive days. In trial one, the mice were placed in the start arm and were allowed to choose one goal arm in 90 s. Once the mice chose to enter one goal arm, the door connecting the goal arm and the center area was shut, and the mice were allowed to explore the chosen arm for 30 s. In trial two, the mice were placed in the start arm and allowed to explore again. Once the mice chose to enter one goal arm, the trial ended. If a mouse entered different goal arms in two trials, a score of one point was recorded. The following formula was used: correct rate % = (total points obtained over 10 days/10) ×100%.

Ex vivo electrophysiology

The mice were anesthetized with isoflurane. The brain was quickly removed from the skull after decapitation, and 300 μm horizontal slices containing the mPFC from cKO

and WT mice were prepared using a Leica LS1200s vibrating microtome in ice-cold oxygenated artificial cerebrospinal fluid (aCSF) containing (in mM) (Sigma, USA) 220 sucrose, 2.5 KCl, 1.3 $CaCl_2$, 2.5 $MgSO_4$, 1 NaH_2PO_4, 26 $NaHCO_3$, and 10 glucose. The recording aCSF contained (in mM) 126 NaCl, 26 $NaHCO_3$, 3.0 KCl, 1.2 NaH_2PO_4, 2.0 $CaCl_2$, 1.0 $MgSO_4$, and 10 glucose. The slices were transferred to a chamber and held at 35°C for 30 min and at room temperature for 1 h. The slices were then kept at room temperature until recording.

A single slice was transferred to the recording chamber that was constantly perfused (~3 mL min^{-1}) with oxygenated aCSF at 35°C. The mPFC pyramidal neurons were viewed under a Zeiss upright microscope (Germany) equipped with a 40× water immersion objective. Recording electrodes with a resistance of 4-8 MΩ were pulled from borosilicate glass capillaries (1.5 mm outside diameter [OD]) using a P97 electrode puller. The internal pipette solution contained (in mM) (Sigma, USA) 125 cesium methanesulfonate, 5 CsCl, 10 HEPES, 0.2 EGTA, 1 $MgCl_2$, 4 Mg-ATP, 0.3 Na-GTP, 10 phosphocreatine and 5 QX314 (pH 7.40, 290 mOsm). The recordings were performed with a HEKA EPC10 amplifier (Germany); the signals were filtered at 5 kHz and digitized at 10 kHz. The action potential amplitude and spiking ability of the recorded cells were assayed by an intracellular injection of step currents (500 ms duration, magnitudes ranging up to 120 pA with steps of 20 pA). We recorded sEPSCs with 20 μM bicuculine (Sigma, USA) added to the cerebrospinal fluid in the voltage clamp (V_{clamp}= −70 mV). mEPSCs were recorded in the presence of 1 μM tetrodotoxin (Aladdin) and 20 μM bicuculine (Sigma, USA).

Proteomics analysis of O-GlcNAcylation

The sample was ground in liquid nitrogen into cell powder and then transferred to a 5-ml centrifuge tube. Protein was extracted and digested by trypsin. After trypsin digestion, the peptide was desalted by a Strata X C18 SPE column (Phenomenex, USA) and vacuum-dried. The peptide was reconstituted in 0.5 M TEAB and processed according to the manufacturer's protocol for the TMT kit. The peptides were combined into 6 fractions and dried by vacuum centrifugation. To enrich the modified peptides, tryptic peptides dissolved in NETN buffer (100 mM NaCl, 1 mM EDTA, 50 mM Tris HCl, 0.5% NP-40, pH 8.0) were incubated with prewashed antibody beads (PTM-952, PTM Bio, China) at 4°C overnight under gentle shaking. Then, the beads were washed and eluted with 0.1% trifluoroacetic acid. Finally, the resulting peptides were desalted with C18 ZipTips (Millipore, Germany) according to the manufacturer's instructions.

The tryptic peptides were dissolved in 0.1% formic acid (solvent A) and directly loaded onto a homemade reversed-phase analytical column (15 cm length, 75 μm i.d.). The gradient comprised increases from 6% to 23% solvent B (0.1% formic acid in 98% acetonitrile) over 26 min, 23% to 35% in 8 min, and 35% to 80% in 3 min and was maintained at 80% for the last 3 min, all at a constant flow rate of 400 nl min^{-1} using an EASY-nLC 1000 UPLC system. The peptides were subjected to an NSI source, followed by tandem mass spectrometry (MS/MS) in a Q ExactiveTM Plus (Thermo) coupled online to ultraperformance liquid chromatography (UPLC). The electrospray voltage applied was 2.0 kV. The m/z scan range was 350 to 1800 for the full scan, and intact peptides were detected in the Orbitrap at a resolution of 70,000. Then, the peptides were selected for MS/MS using the NCE setting of 28, and the fragments were detected in the Orbitrap at a resolution of 17,500. A data-dependent procedure alternated between one MS scan followed by 20 MS/MS

scans with 15.0 s dynamic exclusion. The automatic gain control (AGC) was set at $5E^4$. The fixed first mass was set as 100 m/z. The resulting MS/MS data were processed using the MaxQuant search engine (v.1.5.2.8). The O-GlcNAc modification sites that could be quantified in both groups were adopted for the differential expression analysis.

Gene Ontology enrichment analysis

The proteins were classified by GO annotation into the following three categories: biological process, cellular compartment and molecular function. For each category, a two-tailed Fisher's exact test was employed to test the enrichment of the differentially modified proteins against all identified proteins. GO terms with a corrected p-value < 0.05 were considered significant.

Pathway enrichment analysis

The KEGG database was used to identify enriched pathways, and a two-tailed Fisher's exact test was employed to test the enrichment of the differentially modified proteins against all identified proteins. Pathways with a corrected p < 0.05 were considered significant. These pathways were classified into hierarchical categories according to the KEGG website.

Enrichment-based clustering

Further hierarchical clustering based on the differentially modified protein functional classification (such as GO, domain, pathway, and complex) was performed. These x values were z-transformed in each functional category. These z-scores were then clustered by one-way hierarchical clustering in Genesis. The cluster membership was visualized by a heatmap using the "heatmap.2" function in the "gplots" R package.

Co-expression network

All differentially expressed modified protein database accessions or sequences were searched against the STRING database version 10.1 to identify protein–protein interactions. We retrieved all interactions with a confidence score ≥ 0.7 (high confidence). The interaction network from STRING was visualized using the "networkD3" R package.

Postmortem brain tissue bioinformatics analysis

The GEO dataset (GSE80655) was downloaded from the Gene Expression Omnibus (GEO) database, which consisted of the middle part of the dorsolateral PFC (DLPFC, Brodmann Area 9, BA9) from 23 MDD patients and 23 healthy controls (Sample SL7804 was excluded for the phenotype information loss). These brain samples were collected under the Brain Donor Program at the University of California, Irvine, Department of Psychiatry and Human Behavior. Total RNA was extracted and sequenced on an Illumina Hi-Seq 2000 with a paired-end 50bp read length. Gene expression data that raw reads counts were downloaded from the GEO database, and then normalized to counts per million (CPM) value by CPM function in R package edgR (version 3.36). The differential expression of *Ogt* gene was calculated by the Mann–Whitney U test. The relative expression of *Ogt* gene in the MDD group was conducted by the control group.

Gene expression microarray dataset GSE54570 was downloaded from the GEO, which consisted of 26 total postmortem tissue from the DLPFC in 13 pairs of human subjects with major depressive disorder (MDD) and non-psychiatric control subjects. They used Affymetrix Human Genome U133 Plus 2.0 platforms (Affymetrix Inc., Santa Clara, CA) to detect. Raw gene expression probe-set signal intensities were extracted and normalized by the microarray pipeline MAAPster

(https://github.com/CCBR/MicroArrayPipeline). Pairwise comparisons were performed with Microarray Suite 5.0. This software assigned a reliable significance level for differential expression between two samples hybridized on separate arrays that was based on multiple probe level data information, and was therefore well suited for matched-pair experimental design.

Supplemental Figures

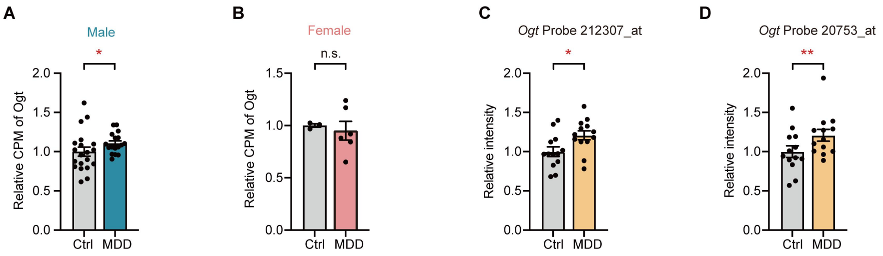

Supplemental Figure 1. *Ogt* was increased in dorsolateral PFC of MDD patients. (A-B) The normalized CPM of *Ogt* transcripts in the DLPFC of male (A) and female (B) MDD patients and healthy controls, the dataset was obtained from GSE80655. (**C-D**) The relative intensity of *Ogt* mRNA probe in the DLPFC of MDD patients and control subjects, the datasets was obtained from GSE54570. All data are presented the mean ± SEM. Mann-Whitney test (**A and B**), Paired *t* test (**C and D**). * $P < 0.05$; ** $P < 0.05$. n.s., no significance. See Source data 2 for statistical details.

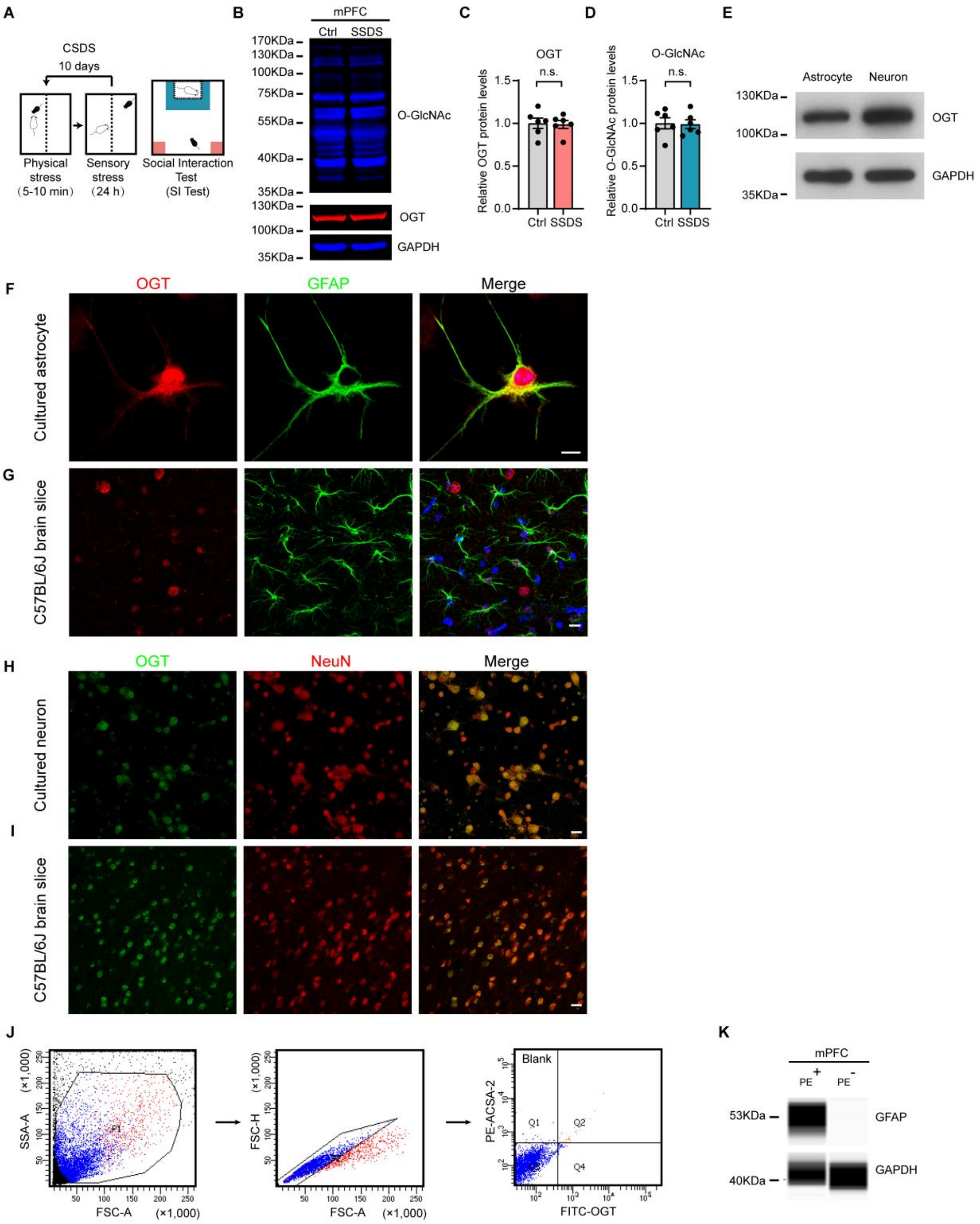

Supplemental Figure 2. OGT is widely expressed in astrocytes and neurons. (**A**) Chronic social defeat stress (CSDS) and social interaction (SI) test protocol. (**B-D**) Western blot images (B) and quantification (C and D) of OGT expression in the mPFC after 3-day subthreshold social defeat stress (SSDS). (**E**) Western blot image of OGT expression in primary cultured astrocytes and neurons. (**F and G**) Double immunofluorescence staining of OGT (red) with GFAP (green) in cultured astrocytes and brain slices of C57BL/6J mice, Scale bar=10 μm. (**H and I**) Double immunofluorescence staining of OGT (green) with NeuN (red) in cultured neurons and brain slices of C57BL/6J mice, Scale bar=20 μm. (**J**) The initial gating strategy was used to define cells in FACS. (**K**) GFAP expression of Astrocyte and Non-astrocyte sorting by FACS in mPFC. All data are presented the mean ± SEM. Two-sided unpaired *t*-test for two-group comparisons (**C and D**). n.s., no significance. See Source data 2 for statistical details.

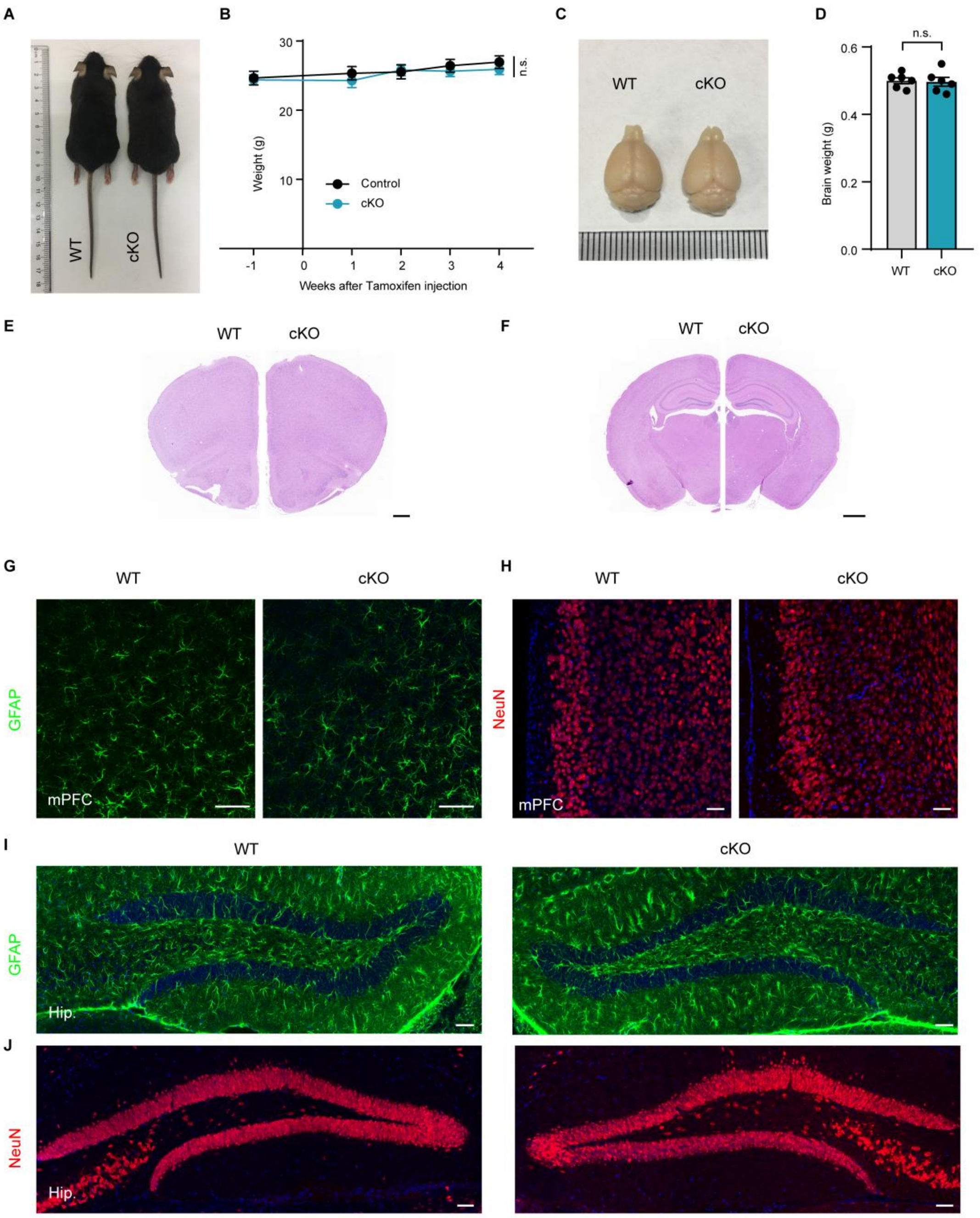

Supplemental Figure 3. Generation of *Fgfr3-iCreER*^{T2}*; OGT*$^{flox/Y}$ mice. (**A and B**) The represented image and quantification of body weight changes after TAM injection for cKO and littermate controls. n=6 (WT), 12 (cKO) mice. (**C and D**) Representative image and the quantification of brain weight change after TAM injection. n=6 (WT), 6 (cKO) mice. (**E and F**) Hematoxylin and eosin (H&E) staining of coronal sections around the mPFC and hippocampus of the brains of cKO and WT mice. Scale bar=500 µm in the mPFC and 1000 µm in the Hip. (**G and I**) Immunofluorescence for GFAP (green) in the mPFC and Hip. Scale bar=50 µm. (**H and J**) Immunofluorescence for NeuN (red) in the mPFC and Hip. Scale bar=50 µm. All data are presented the mean ± SEM. Two-sided unpaired *t*-test for two-group comparisons (**D**), and Two-way ANOVA with Bonferroni's multiple comparisons test (**B**). n.s., no significance. See Source data 2 for statistical details.

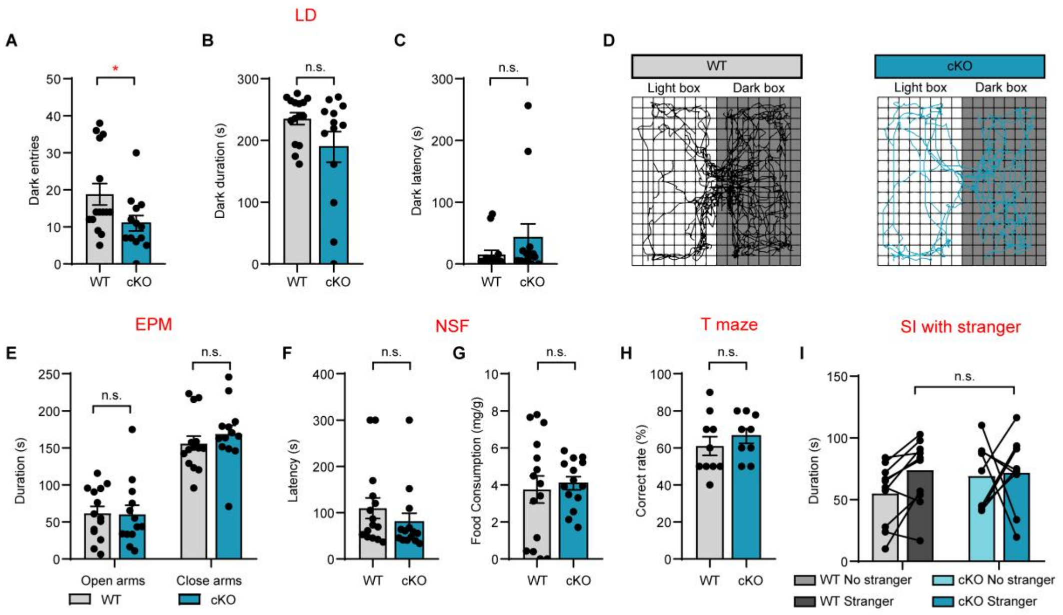

Supplemental Figure 4. Behavioral tests of astrocytic OGT deletion mice and WT controls. (**A-D**) Light-dark box (LDB) test and represented tracks for two groups. n=15 (WT), 13 (cKO). (**E**) Elevated plus maze (EPM) for the cKO and control mice. n=14 (WT), 13 (cKO). (**F and G**) Novelty suppressed feeding (NSF) test for cKO and WT mice. n=15 (WT), 14 (cKO). (**H**) T maze for the cKO and WT mice. n=10 (WT), 9 (cKO) mice. (**I**) Social novelty with a stranger for two groups. n=11 (WT), 9 (cKO) mice. All data are presented the mean ± SEM. Two-sided unpaired *t*-test for two-group comparisons (**A-H**), and Two-way ANOVA with Bonferroni's multiple comparisons test (**I**). * $P< 0.05$; n.s., no significance. See Source data 2 for statistical details.

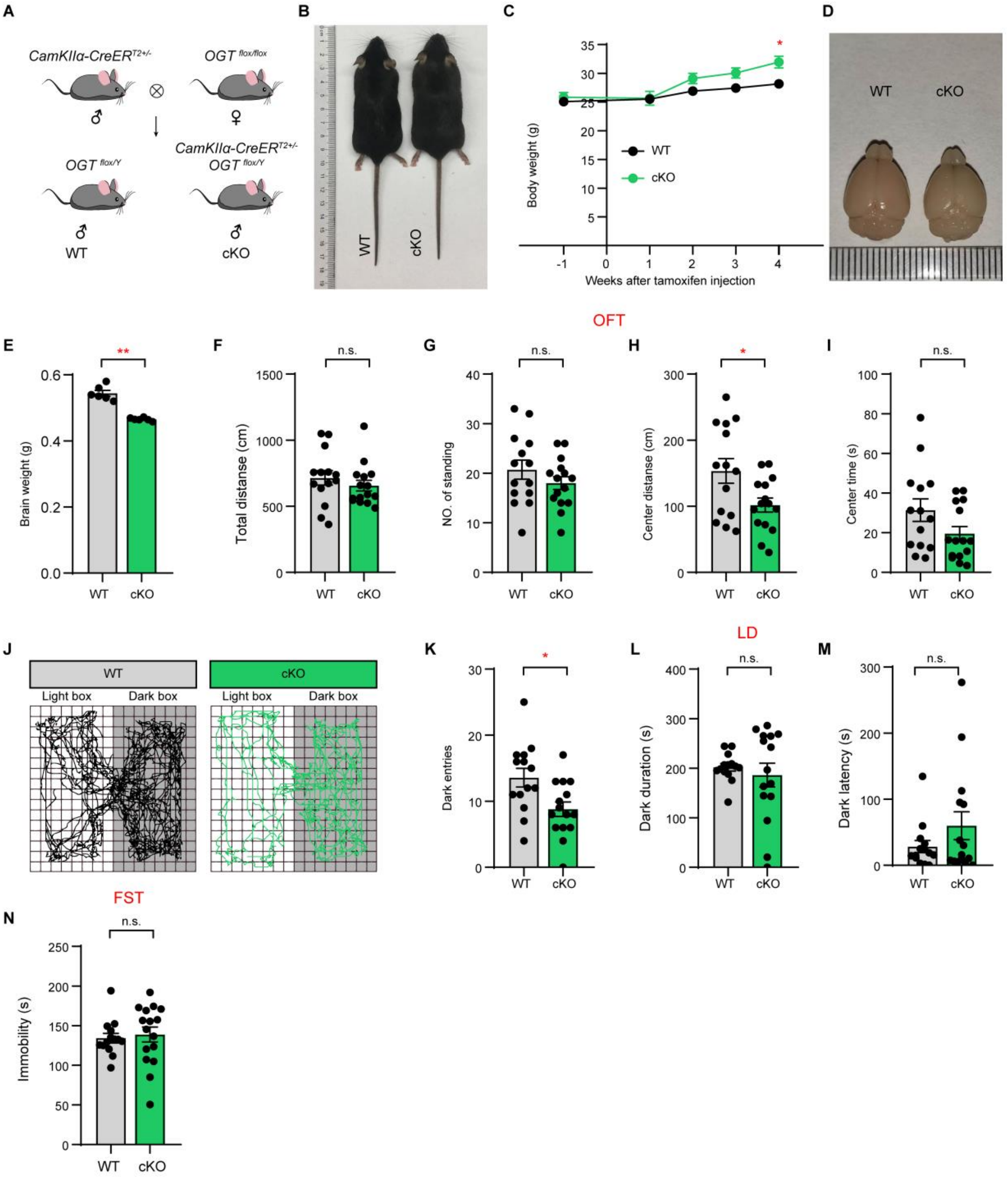

Supplemental Figure 5. Behavioral tests of neuron-specific OGT cKO and WT mice. (**A**) Generation of neuron-specific OGT cKO mice by crossing *OGT$^{flox/flox}$* lines with *CaMK Ⅱα-iCreER^{T2}* mouse lines. (**B and C**) Representative image (B) and the quantification (C) of body weight changes after TAM injection. n=14 (WT), 10 (cKO) mice. (**D and E**) Representative image (D) and quantification (E) of brain weight changes after TAM injection. n=6 (WT), 6 (cKO). (**F-I**) OFT for the cKO and WT mice. n=14 (Ctrl.), 15 (cKO). (**J-M**) LDB test and represented tracks for two groups. n=14 (WT), 15 (cKO) mice. (**N**) Immobility times of *CaMK Ⅱα-iCreER^{T2}; OGT$^{flox/Y}$* (cKO) and WT mice in FST. n=14 (WT), 16 (cKO) mice. All data are presented the mean ± SEM. Two-sided unpaired *t*-test (**E-I, K-N**) for two-group comparisons, and Two-way ANOVA with Bonferroni's multiple comparisons test (**C**). * *P*< 0.05; ** *P*< 0.01; n.s., no significance. See Source data 2 for statistical details.

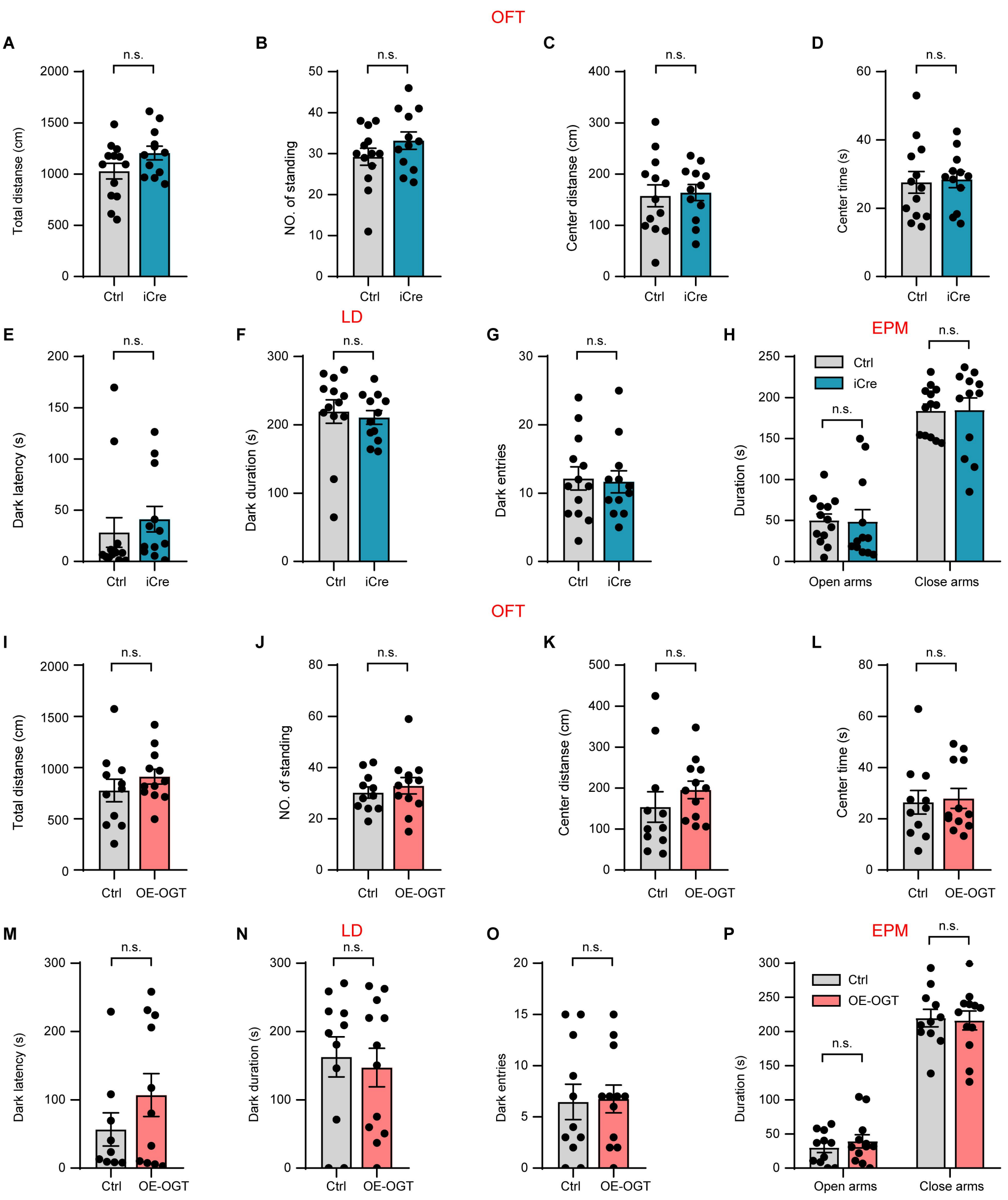

Supplemental Figure 6. Behavioral test results of astrocyte-specific OGT knockdown or overexpression in the mPFC. (**A-D**) OFT results for astrocyte-specific OGT knockdown mice. n=13 (Ctrl.), 12 (iCre) mice. (**E-G**) LDB results for astrocyte-specific OGT knockdown mice. n=13 (Ctrl.), 12 (iCre) mice. (**H**) EPM results for astrocyte-specific OGT knockdown mice. n=13 (Ctrl.), 12 (iCre) mice. (**I-L**) OFT results for astrocyte-specific OGT overexpression mice. n=11 (Ctrl.), 12 (OE) mice. (**M-O**) LDB results for astrocyte-specific OGT overexpression mice. n=11 (Ctrl.), 11 (OE) mice. (**P**) EPM results for astrocyte-specific OGT overexpression mice. n=10 (Ctrl.), 11 (OE) mice. All data are presented the mean ± SEM. Two-sided unpaired *t*-test for two-group comparisons (**A-P**). See Source data 2 for statistical details.

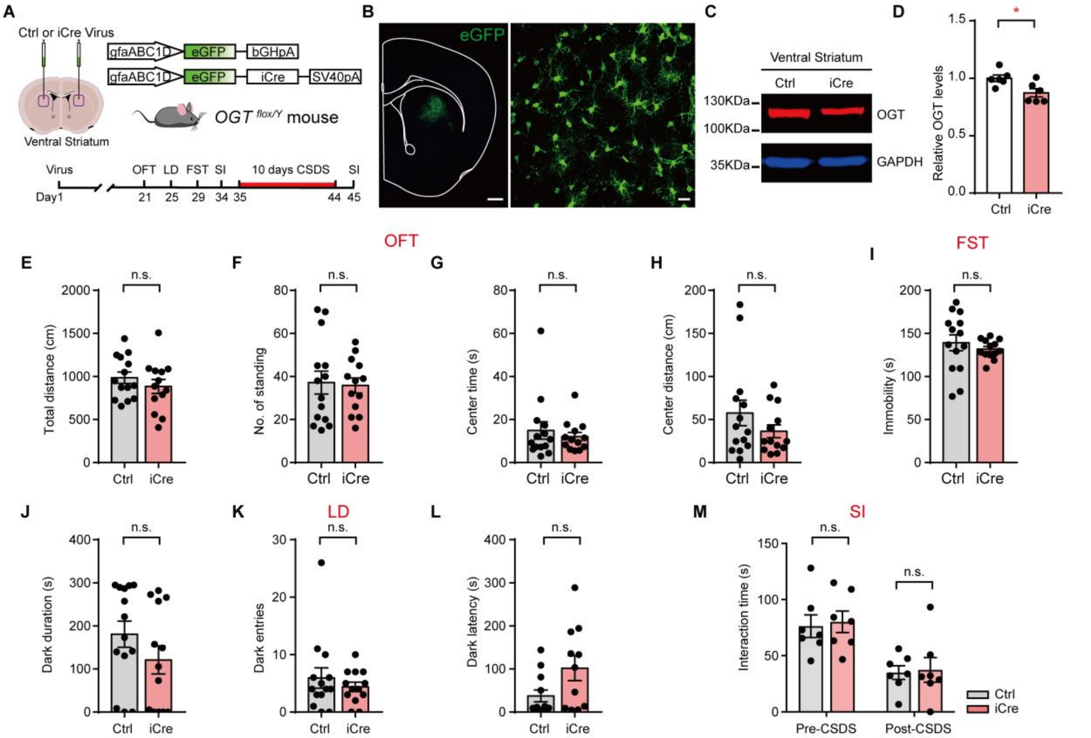

Supplemental Figure 7. Astrocyte-specific knockdown of OGT in the Ventral Striatum did not alter depressive-like behaviors. (**A**) Schematic of the AAV vectors engineered to specifically knockdown astrocytic OGT in the Ventral Striatum and paradigms of behavioral tests. (**B**) Representative images of injection sites in the Ventral Striatum of *OGT*$^{flox/Y}$; scale bar=500 μm. Left, magnified view of left image, scale bar=20 μm. Right. (**C and D**) Western blot images (C) and quantification (D) of OGT in the Ventral Striatum of *OGT*$^{flox/Y}$ mice. n=6 (Ctrl), 6 (iCre) mice. (**E-H**) OFT results for astrocyte-specific OGT knockdown in the Ventral Striatum of *OGT*$^{flox/Y}$ mice, n=14 (Ctrl), 13 (iCre) mice. (**I**) Immobility time in the FST, n=14 (Ctrl), 13 (iCre) mice. (**J-L**) LD results for astrocyte-specific OGT knockdown in the Ventral Striatum of *OGT*$^{flox/Y}$ mice, n=14 (Ctrl), 13 (iCre) mice. (**M**) Social interaction time in the presence of social targets before and after CSDS. n=7 (Ctrl), 7 (iCre) mice. All data are presented the mean ± SEM. Two-sided unpaired *t*-test (**D-F, I and K**) or Mann Whitney test (**G, H, J and L**) for two-group comparisons. Two-way ANOVA with Bonferroni's multiple comparisons test (**M**). * $P< 0.05$; n.s. no significance. See Source data 2 for statistical details.

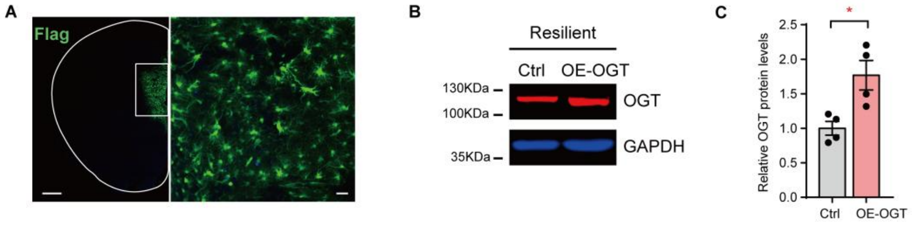

Supplemental Figure 8. OGT protein levels were increased in the mPFC of resilient mice after virus expression. (**A**) Representative images of injection sites in the mPFC of $OGT^{flox/Y}$; scale bar=500 µm. Right, magnified view of left image, scale bar=20 µm. Left. (**B and C**) Western blot images (B) and quantification (C) of OGT in the mPFC of $OGT^{flox/Y}$ mice. n=4 (Ctrl), 4 (OE) mice. All data are presented the mean ± SEM. Two-sided unpaired *t*-test (**C**) for two-group comparisons. * $P< 0.05$. See Source data 2 for statistical details.

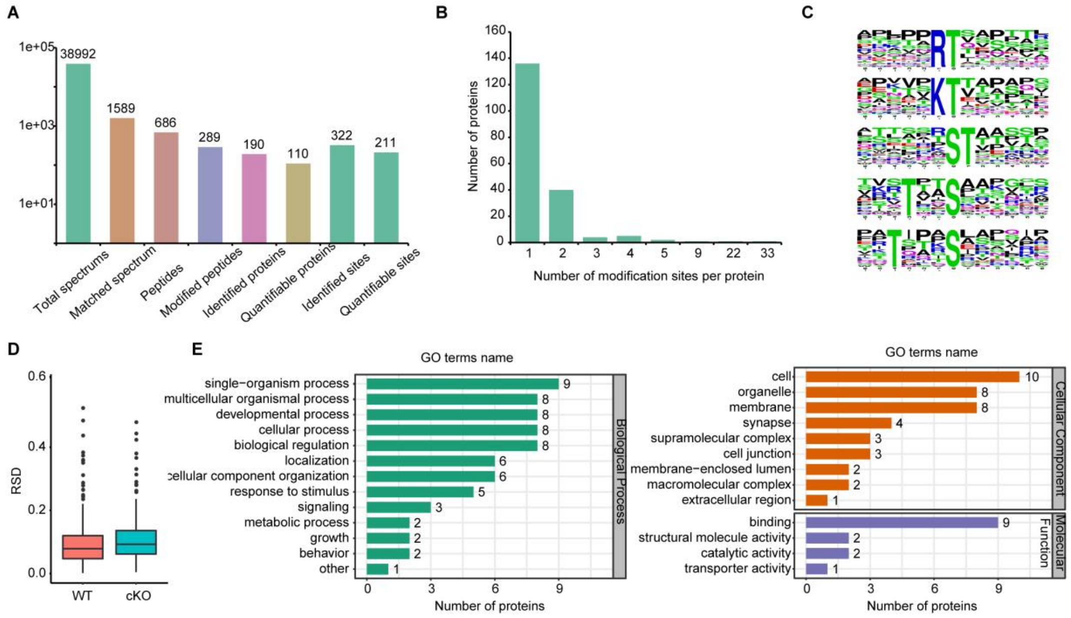

Supplemental Figure 9. Basic analysis of O-GlcNAcylation proteins in the mPFC of astrocytic OGT cKO and WT mice. (**A**) Basic information of MS data. (**B**) Number of O-GlcNAc modification sites per protein. (**C**) Sequence motif analysis of protein O-GlcNAcylation in this study. (**D**) Relative standard deviation (RSD) in WT and cKO mice. (**E**) GO classification of differentially modified proteins, including the biological process, cellular compartment and molecular function categories. Two-tailed Fisher's exact test and corrected $P<0.05$ were considered significant.

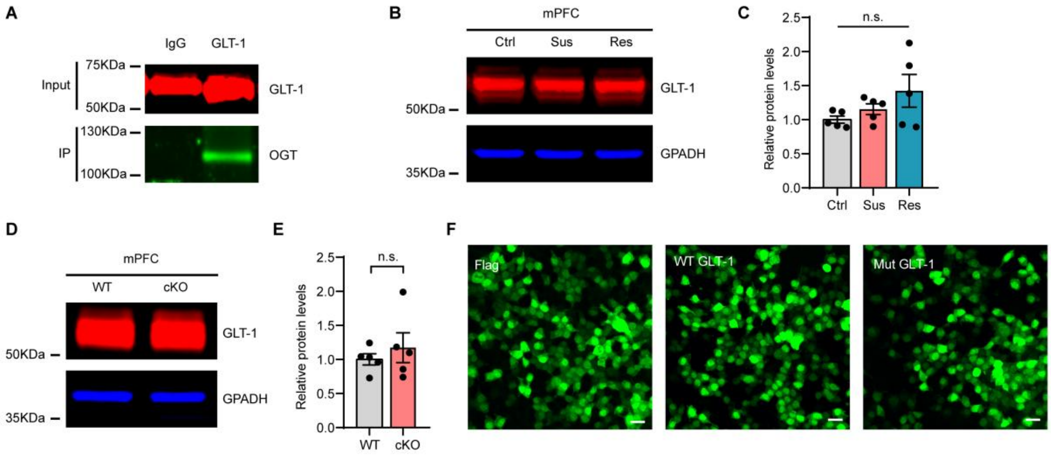

Supplemental Figure 10. The total expression of GLT-1 was not changed in the mPFC. (**A**) Immunoprecipitation and Western blotting were used to check the interaction between GLT-1 and OGT. (**B**) Representative images of H293T cells transfected with the mutant GLT-1, WT GLT-1 and blank plasmids. (**C and D**) Western blot images (C) and quantification (D) of GLT-1 expression in the mPFC after the CSDS paradigm. The blots were cropped according to their molecular weight, and tissue levels of GAPDH were used as a loading control. Protein expression was normalized to the control levels. n= 5 in each group. (**E and F**) Western blot images (E) and quantification (F) of GLT-1 expression in the mPFC of WT and cKO mice. n= 5 in both groups. All data are presented the mean ± SEM. Two-sided unpaired *t*-test for two-group comparisons (**F**), One-way with Bonferroni's multiple comparisons test (**D**). n.s., no significance. See Source data 2 for statistical details.

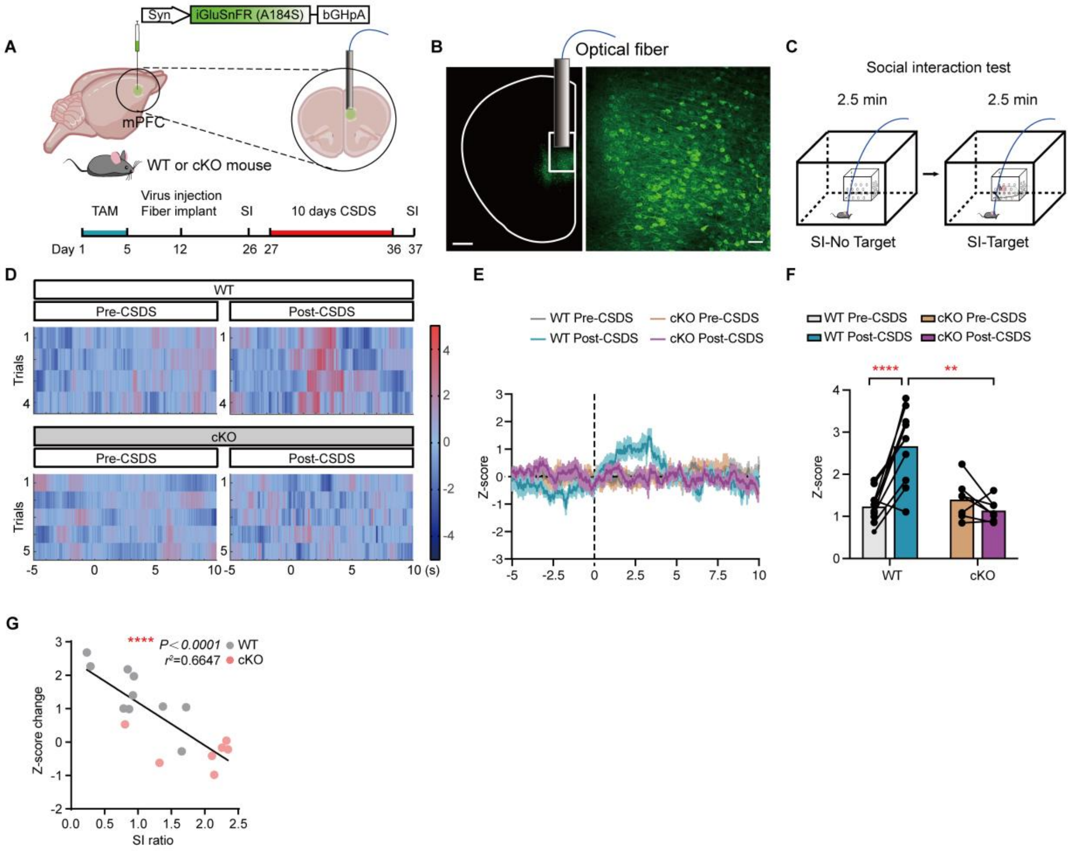

Supplemental Figure 11. Astrocytic OGT regulates glutamate response in social interaction test. (**A and B**) Schematic of the AAV vectors engineered to express glutamate sensor (iGluSnFR A184S) under a *syn* promoter (A), and representative image of injection sites in the mPFC (B); scale bar=500 µm. Right, magnified view of left image, scale bar=50 µm. (**C**) Paradigms of photometry recordings during the social interaction test (SI). (**D**) Representative heatmaps of z-score changes over all trials from single mice. (**E and F**) Time course of average iGluSnFR transient z-scores event-locked to social interaction (**E**) and quantification (**F**) of the average peak z-score during social interaction. n=10 (WT), 7 (cKO) mice. (**G**) The correlation analysis between the changed z-score and SI ratio, r^2=0.6647, P< 0.0001. All data are presented the mean ± SEM. Two-way ANOVA with Bonferroni's multiple comparisons test (**F**). ** P< 0.01; **** P< 0.0001; n.s., no significance. See Source data 2 for statistical details.

Unedited Western blot images

Full unedited Western blots for Figure 1

mPFC, C57 after CSDS

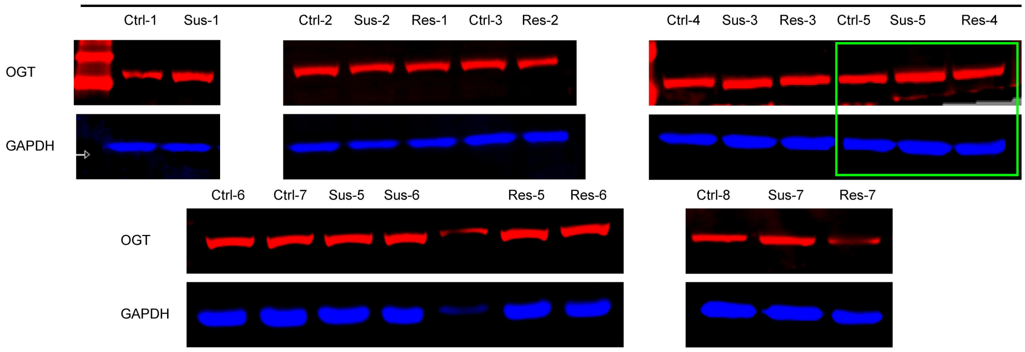

Striatum, C57 after CSDS

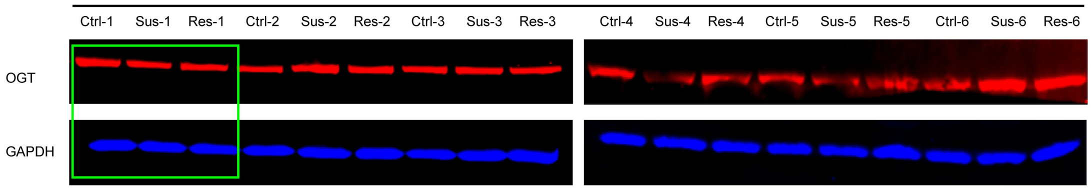

NAc, C57 after CSDS

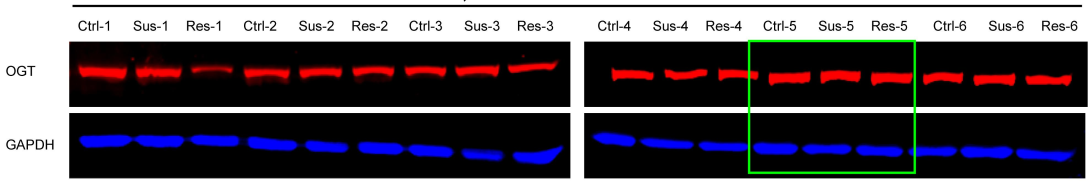

Hip, C57 after CSDS

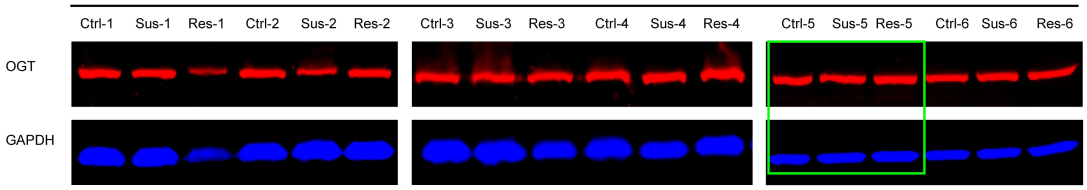

Amy, C57 after CSDS

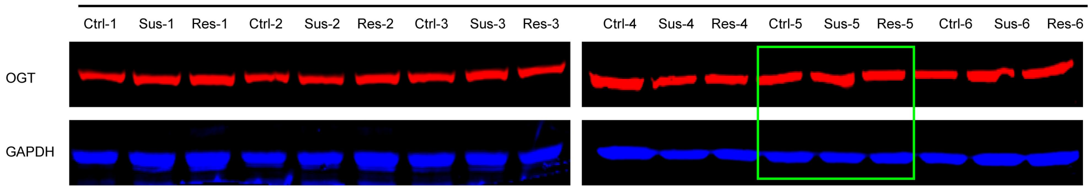

DRN, C57 after CSDS

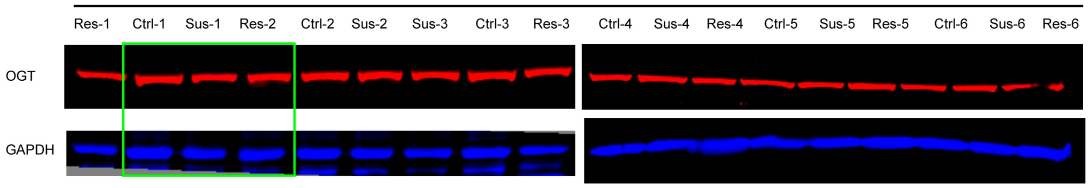

Full unedited Western blots for Figure 1

O-GlcNAc, mPFC, C57 after CSDS

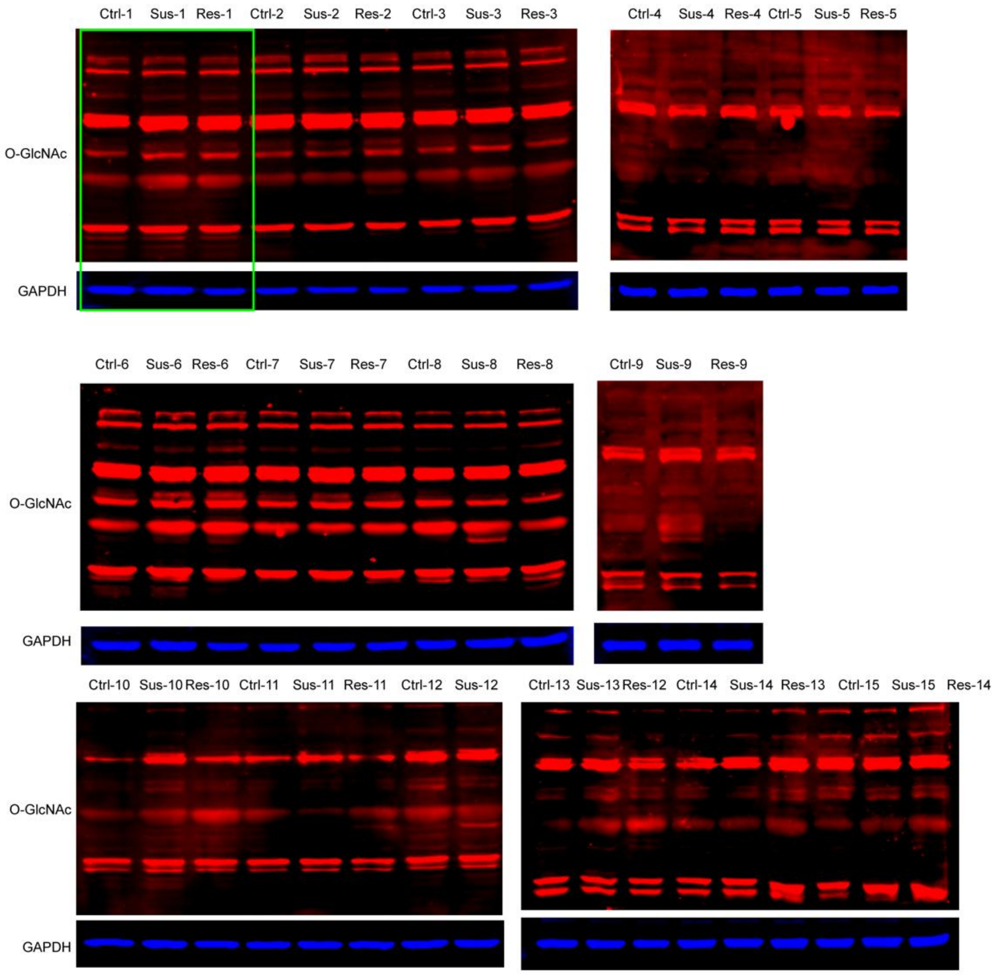

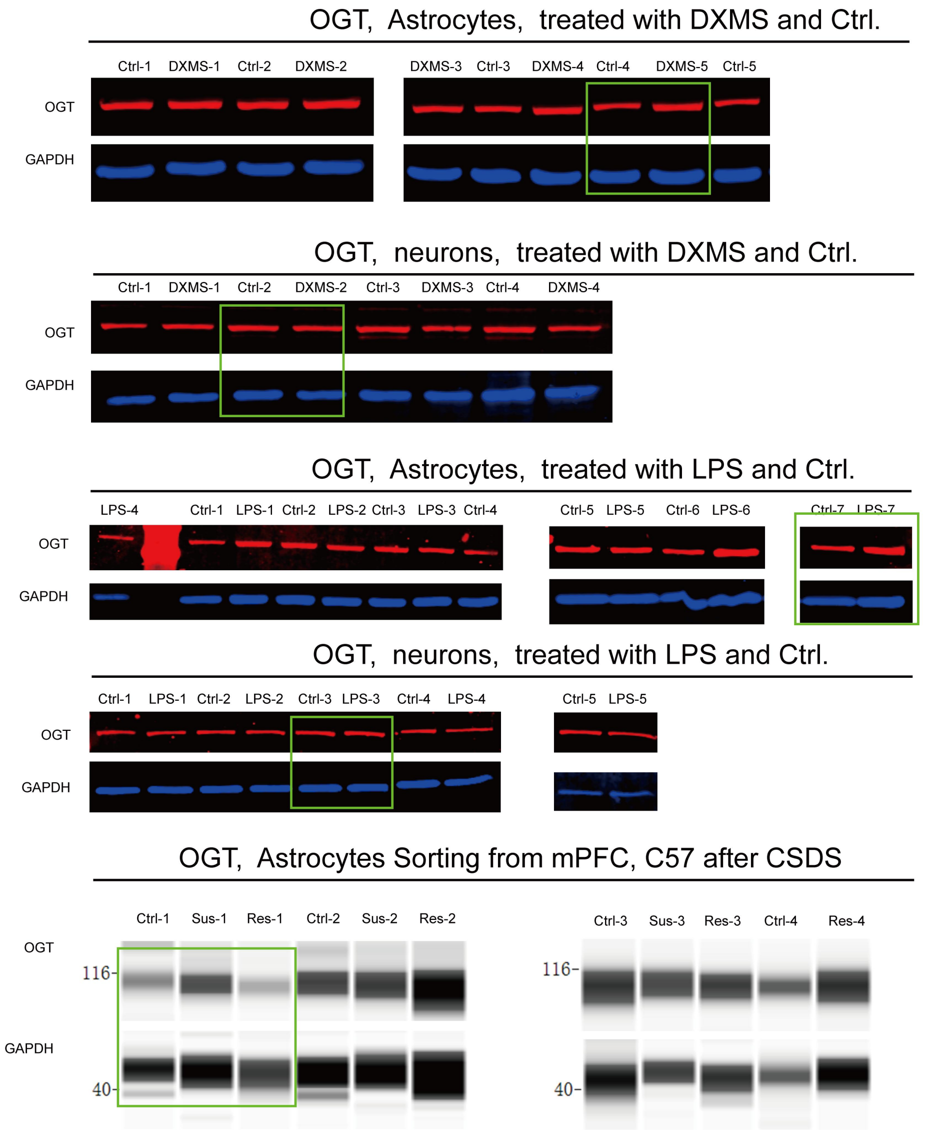
OGT, Astrocytes, treated with DXMS and Ctrl.
Ctrl-1 DXMS-1 Ctrl-2 DXMS-2 DXMS-3 Ctrl-3 DXMS-4 Ctrl-4 DXMS-5 Ctrl-5
OGT
GAPDH
OGT, neurons, treated with DXMS and Ctrl.
Ctrl-1 DXMS-1 Ctrl-2 DXMS-2 Ctrl-3 DXMS-3 Ctrl-4 DXMS-4
OGT
GAPDH
OGT, Astrocytes, treated with LPS and Ctrl.
LPS-4 Ctrl-1 LPS-1 Ctrl-2 LPS-2 Ctrl-3 LPS-3 Ctrl-4 Ctrl-5 LPS-5 Ctrl-6 LPS-6 Ctrl-7 LPS-7
OGT
GAPDH
OGT, neurons, treated with LPS and Ctrl.
Ctrl-1 LPS-1 Ctrl-2 LPS-2 Ctrl-3 LPS-3 Ctrl-4 LPS-4 Ctrl-5 LPS-5
OGT
GAPDH
OGT, Astrocytes Sorting from mPFC, C57 after CSDS
Ctrl-1 Sus-1 Res-1 Ctrl-2 Sus-2 Res-2
Ctrl-3 Sus-3 Res-3 Ctrl-4 Res-4
OGT
GAPDH
116-
40-

Full unedited Western blots for Figure 4

OGT, mPFC, *OGT* $^{loxp/loxp}$ mice with gfaABC1D-iCre virus

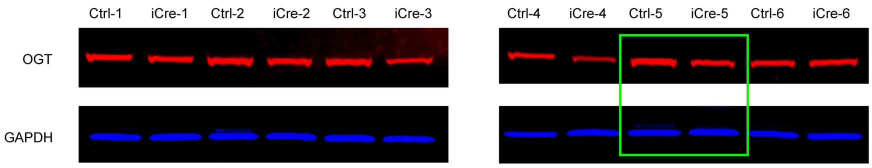

OGT, mPFC, *Fgfr-creER* ^{T2} mice with Dio-OGT virus

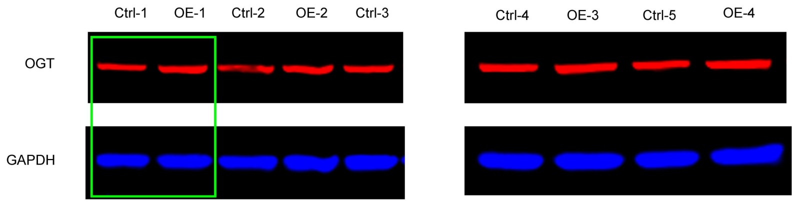

Full unedited Western blots for Figure 6

GLT-1/O-GlcNAc, Cultured astrocyte, Treated with OSMI-1

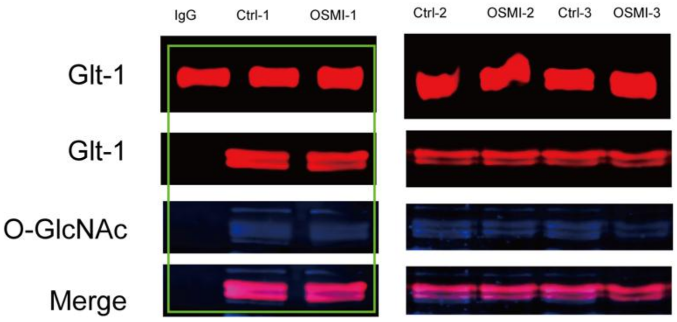

GLT-1/O-GlcNAc, mPFC, C57 after CSDS

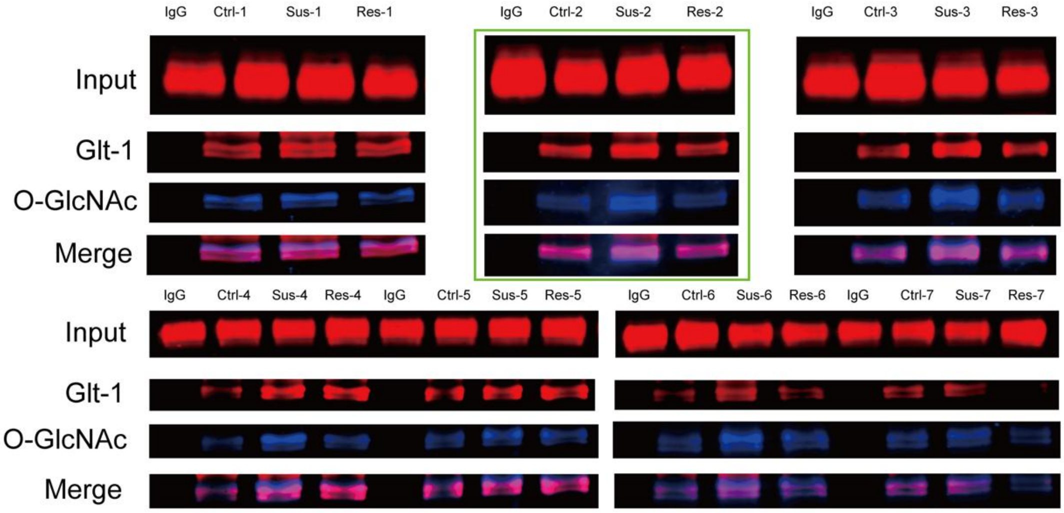

GLT-1/O-GlcNAc, mPFC, Fgfr-OGT cKO mice

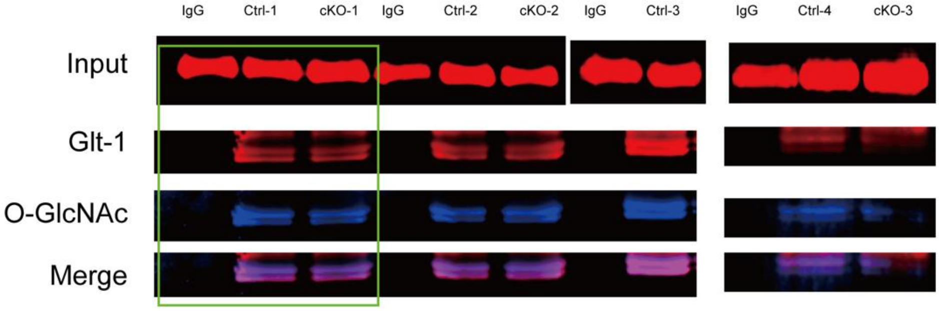

Full unedited Western blots for Figure S1

OGT, Primary cultured astrocytes and neurons

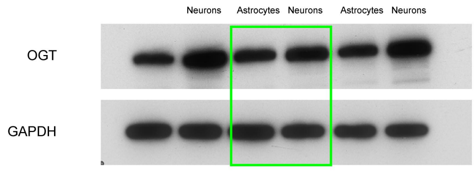

OGT/O-GlcNAc, mPFC, C57 after 3-day SSDS

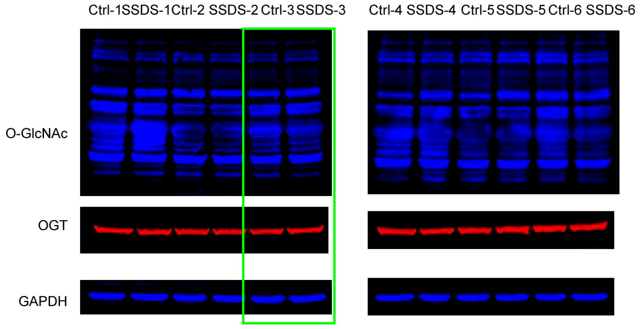

Full unedited Western blots for Figure S6

OGT, Ventral Striatum, *OGT* $^{loxp/loxp}$ mice with gfaABC1D-iCre virus

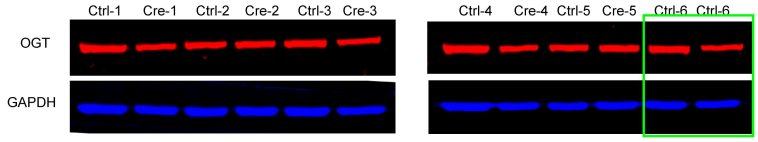

Full unedited Western blots for Figure S7

OGT, mPFC, *Fgfr-creER* ^{T2} mice with Dio-OGT virus after CSDS

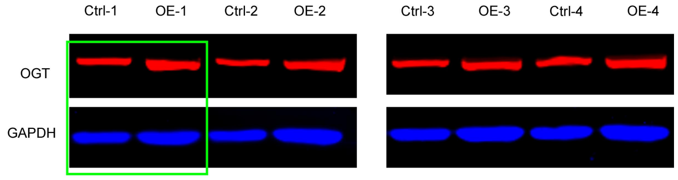

Full unedited Western blots for Figure S9

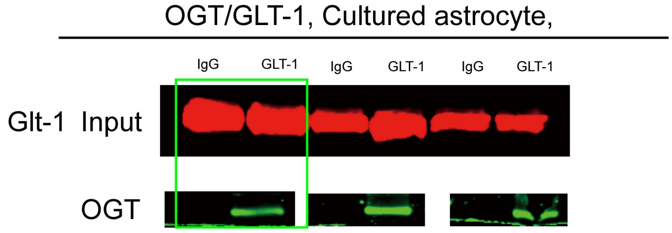

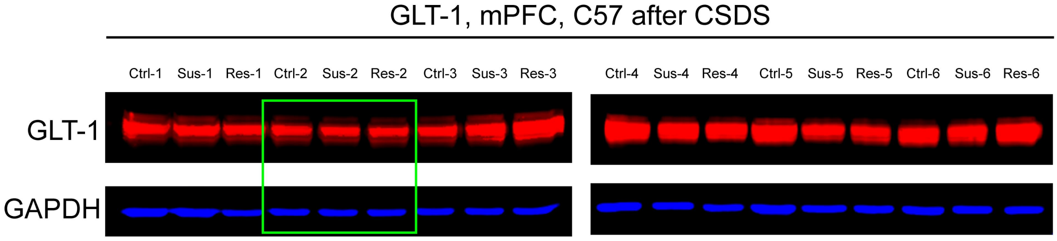

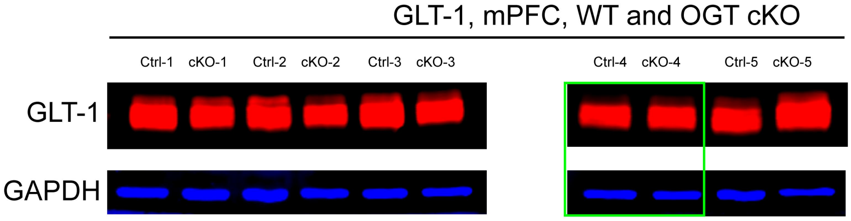

Supplement: Supplemental data [file jci-133-160016-s053.pdf]
