# Supplementary material for: O-GlcNAc transferase in astrocytes modulates depression-related stress susceptibility through glutamatergic synaptic transmission
Source: J Clin Invest. 2023 Apr 3;133(7):e160016. doi: 10.1172/JCI160016 (PMC10065078; doi:10.1172/JCI160016)
Supplement: Supplemental tables 1-3 [file jci-133-160016-s056.pdf]

Supplementary Table 1. Participants' demographics and clinical characteristics

Characteristic	Male		
	Healthy control	MDD	*p* value
n	38	33	
Age (Years)	26.18 ± 0.7991	28.27 ± 1.852	0.8971
Family history	1/38	13/33	0.001
First episode (%)	NA	10/33	
On-set age (years)	NA	23.28	
Disease duration (months)	NA	59.53	
Bipolar depression	NA	10/33	
	Female		
	Healthy control	MDD	
n	39	37	
Age (Years)	24.79 ± 0.4890	26.08 ± 1.369	0.8092
Family history	2/39	11/37	0.0056
First episode (%)	NA	7/37	
On-set age (years)	NA	22.72	
Disease duration (months)	NA	68.52	
Bipolar depression	NA	11/37	

NA, Not Available; All data are all presented as the mean ± SEM.

Supplementary Table 2. Male participants’ depression symptom characteristics

Characteristic	Male		
HAMD-Diurnal variation	Healthy control	MDD	*p* value
n	38	33	
SCL-90	27.00 ± 1.73	180.80 ± 12.51	<0.0001
HAMD-24	3.29 ± 0.2208	36.88 ± 0.4214	<0.0001
HAMD-Anxiety	0.63 ± 0.1027	3.76 ± 0.2821	<0.0001
HAMD-Weight	0.21 ± 0.0670	0.97 ± 0.1409	<0.0001
HAMD-Cognition	0.24 ± 0.0879	9.76 ± 0.4905	<0.0001
HAMD-Diurnal variation	0.21 ± 0.0769	1.06 ± 0.1372	<0.0001
HAMD-Retardation	0.26 ± 0.0816	6.88 ± 0.3131	<0.0001
HAMD-Sleep	0.66 ± 0.1149	5.18 ± 0.2016	<0.0001
HAMD-Hopeless	0.61 ± 0.1036	7.85 ± 0.2987	<0.0001
HAMD-Suicide	0	1.49 ± 0.1385	<0.0001

SCL-90: Symptom Check List 90; HAMD-24: 24-item Hamilton Depression Scale; HAMD-Anxiety: Hamilton Depression Scale-Anxiety/somatization factor; HAMD-Weight: Hamilton Depression Scale-Loss of weight factor; HAMD-Cognition: Hamilton Depression Scale-Cognitive factor; HAMD-Diurnal Variation: Hamilton Depression Scale-Diurnal variation factor; HAMD-Retardation: Hamilton Depression Scale-Retardation factor; HAMD-Sleep: Hamilton Depression Scale-Sleep disorder factor; HAMDHopeless: Hamilton Depression Scale-Hopeless, helpless, and worthless factor; HAMD-Suicide: Hamilton Depression Scale-Suicide factor. All data are all presented as the mean ± SEM.

Supplementary Table 3. Female participants' depression symptom characteristics

Characteristic	Male		
HAMD-Diurnal variation	Healthy control	MDD	*p* value
n	39	37	
SCL-90	25.31 ± 1.272	186.4 ± 12.37	<0.0001
HAMD-24	3.10 ± 0.2888	37.03 ± 0.3818	<0.0001
HAMD-Anxiety	0.69 ± 0.1049	4.37 ± 0.2757	<0.0001
HAMD-Weight	0.13 ± 0.0655	1.03 ± 0.1162	<0.0001
HAMD-Cognition	0.41 ± 0.1021	10.37 ± 0.4140	<0.0001
HAMD-Diurnal variation	0.36 ± 0.1005	1.26 ± 0.1111	<0.0001
HAMD-Retardation	0.33 ± 0.0925	5.61 ± 0.2836	<0.0001
HAMD-Sleep	0.85 ± 0.1401	4.84 ± 0.1942	<0.0001
HAMD-Hopeless	0.33 ± 0.0995	7.71 ± 0.2870	<0.0001
HAMD-Suicide	0	1.87 ± 0.1776	<0.0001

SCL-90: Symptom Check List 90; HAMD-24: 24-item Hamilton Depression Scale; HAMD-Anxiety: Hamilton Depression Scale-Anxiety/somatization factor; HAMD-Weight: Hamilton Depression Scale-Loss of weight factor; HAMD-Cognition: Hamilton Depression Scale-Cognitive factor; HAMD-Diurnal Variation: Hamilton Depression Scale-Diurnal variation factor; HAMD-Retardation: Hamilton Depression Scale-Retardation factor; HAMD-Sleep: Hamilton Depression Scale-Sleep disorder factor; HAMDHopeless: Hamilton Depression Scale-Hopeless, helpless, and worthless factor; HAMD-Suicide: Hamilton Depression Scale-Suicide factor. All data are all presented as the mean ± SEM.
